# Comparative analysis of surgical and endovascular aneurysm repair in subarachnoid hemorrhage: a single-center study with 1,171 patients

**DOI:** 10.1007/s00701-025-06670-w

**Published:** 2025-09-13

**Authors:** Per Kristian Eide, Wilhelm Sorteberg, Are H. Pripp, Pål A. Rønning, Angelika G. Sorteberg

**Affiliations:** 1 Department of Neurosurgery, Oslo University Hospital - Rikshospitalet, Oslo, Norway; 2 Institute of Clinical Medicine, Faculty of Medicine, University of Oslo, Oslo, Norway; 3 KG Jebsen Centre for Brain Fluid Research, University of Oslo, Oslo, Norway; 4 Oslo Centre of Biostatistics and Epidemiology, Research Support Services, Oslo University Hospital, Oslo, Norway; 5 Faculty of Health Sciences, Oslo Metropolitan University, Oslo, Norway

**Keywords:** Aneurysmal subarachnoid hemorrhage, Endovascular treatment, Surgical clipping, Mortality, Modified rankin score, Outcome

## Abstract

**Background:**

To compare surgical and endovascular therapy (EVT) approaches to aneurysm repair in all aneurysmal subarachnoid hemorrhage (aSAH) patients treated within our institution over a 12-year period from 2011 to 2022.

**Methods:**

The study comprised a retrospective analysis of prospectively collected data extracted from a hospital quality registry that we established in 2011, containing comprehensive information about all patients treated for aSAH. We included SAH patients within the institution's catchment area who underwent surgical or endovascular aneurysm repair. Exclusion criteria involved patients from external regions, those treated at other institutions, no aneurysm repair performed, or instances undergoing a combination of surgery and EVT. Pretreatment data encompassed the clinical condition at admission, comorbidity, radiological details, aneurysm characteristics, and duration between the bleed and aneurysm repair. Mortality was primary outcome measure; secondary outcome included modified Rankin Score after approximately six months.

**Results:**

The study encompassed 1,171 patients (65% women and 35% men) undergoing aneurysm repair from 2011 to 2022. Admission data revealed 31.1% in Hunt-Hess grade 4–5. Surgical repair was performed in 573 (48.9%) patients, and EVT in 598 (51.1%) patients. Pretreatment information was comparable for both groups. Kaplan–Meier survival curves demonstrated lower mortality in the surgical than the EVT group (*P* = 0.023; Log-rank test) over the 12-year period. The 1-year, 5-year, and 10-year mortality rates were 12.4%, 19.5%, and 27.7% for the surgery group, and 18.7%, 25.2%, and 31.7% for the EVT group, respectively. Modified Rankin Score was worse for EVT. There was lower mortality in surgical than EVT groups in patients treated for anterior communicating artery (ACOM, *n* = 420) and posterior communicating artery (PCOM, *n* = 177) aneurysms. Shorter time to aneurysm repair and more extensive cerebrospinal fluid (CSF) drainage characterized the surgery group.

**Conclusions:**

Mortality was lower in surgical patients. Plausible explanations are the maintenance of surgical skills and prompt reduction of intracranial pressure.

**Supplementary Information:**

The online version contains supplementary material available at 10.1007/s00701-025-06670-w.

## Introduction

Subarachnoid hemorrhage (SAH) poses a significant threat to life, with approximately 8 out of 10 cases attributed to the rupture of a cerebral aneurysm [2, 13]. Timely intervention for the ruptured aneurysm is a crucial aspect of patient management, with options including endovascular techniques or open microsurgical procedures, commonly known as clip surgery [11, 17]. Both approaches are concurrently employed, with a prevailing inclination towards endovascular modalities in many regions [1, 18]. The endorsement of endovascular therapy (EVT) as superior to microsurgical methods, particularly emphasized after the ISAT trial [16], remains a subject of debate due to limitations within the trial, such as variations in the study groups, including differences in the timing of aneurysm repair [23].

Over the past two decades, there has been a global trend favoring EVT in aneurysmal SAH (aSAH), though differences in practice exist [1, 3, 9, 19]. Such shifts in medical practices necessitate continuous monitoring of outcome results [14]. In the context of ruptured aneurysm repair, this surveillance is vital to assist physicians, patients, and their families in making informed decisions.

Beginning in 1992, our institution was the first Nordic center to introduce coil treatment of intracranial aneurysms. Performing yearly 150–200 aneurysm procedures, our experience during the following decade made us establish an institutional protocol regarding aneurysm repair in aSAH [20]. With only minor alterations in the protocol later [5], this has led to about the same frequency for both modalities and our institution remaining a high volume center with regard to both clip surgery and EVT in aSAH [20]. This represents an advantageous prerequisite to compare outcomes of the two methods in aSAH patients within our catchment area of 3.2 million inhabitants where our hospital is the sole center treating all aSAH patients.

Since 2011, we established a hospital quality registry for prospective sampling of information from aSAH patients, including information about outcome of treatment. Based on the data collected in this registry, the objective of the present study was to assess and compare mortality rates (primary outcome) and functional (secondary) outcome in aSAH patients that underwent aneurysm repair with clip surgery or EVT at our institution during the 12-year period 2011–2022.

## Materials and methods

### Patient material

The study analyzed data that were prospectively collected within the Neurovascular-CSF Quality Registry (Registration Number: 2011–6692), which since 2011 has documented pre-, per and post-treatment information about all patients treated for aSAH at our institution, including information about outcome.

Inclusion criteria for the present study comprised: i) Patients within the catchment area of our institution who underwent aneurysm repair for aSAH in our department over the 12-year period from January 1 st, 2011, to December 31 st, 2022; for all subjects the information was collected prospectively. ii) Aneurysm repair performed through either surgical intervention or endovascular techniques.

Exclusion criteria were defined as follows: i) Aneurysm repair for aSAH conducted at other institutions with patients subsequently transferred to our institution for intensive care management. ii) Aneurysm repair carried out at our institution on SAH patients from other countries or from other regions within our country, thereby hindering follow-up. iii) Patients where no aneurysm repair occurred, either due to the inability to identify cerebral aneurysms or because extremely poor clinical condition did not warrant aneurysm repair. iv) Aneurysms treated using a combination of surgical and endovascular modalities. v) SAH from aneurysm on a feeder artery to an arterio-venous malformation. vi) Patients listed in the patient registry with no SAH, representing non-SAH aneurysm repair.

Our perioperative management strategy has been described in detail before [5, 20, 21]. The institutional protocol involves collaborative decision-making between the interventional radiologist and the vascular neurosurgeon regarding type of aneurysm repair. Consequently, the consensus-based approach was employed for aneurysm repair in the present cases. The personnel involved were largely the same during the study period.

### Aneurysm categories

The aneurysms were categorized as follows: ***ACOM*** (anterior communicating cerebral artery aneurysms, encompassing the A1 segment of the anterior cerebral artery—ACA), ***BA*** (basilar artery aneurysms, including the BA top, BA-P1 site, mid BA, and the BA-superior cerebellar artery—SCA confluence), ***BBA-ICA*** (blood-blister aneurysms at the internal carotid artery—ICA), ***ICA*** (ICA aneurysms, including ICA top aneurysms), ***MCA*** (middle cerebral artery aneurysms), ***PCOM*** (posterior communicating artery aneurysms), ***Pericallosal*** (pericallosal artery aneurysms, covering the segments of ACA distal to ACOM), and ***VA*** (vertebral artery aneurysms, including aneurysms at the posterior and anterior inferior cerebellar artery).

### Variables

The prospectively collected pre-treatment information encompassed: i) Demographic details (age, sex). ii) Co-morbidity (arterial hypertension and diabetes mellitus) and smoking habits. iii) Clinical condition prior to aneurysm repair (Hunt & Hess grade [12]). iv) Radiological details regarding the hemorrhage (presence and size of intracerebral hemorrhage, modified Fisher grade of subarachnoid bleed [8], aneurysm size and location, LeRoux grade of ventricular bleed [15], and presence of acute subdural hematoma). v) Logistic information (time from ictus to aneurysm repair, time from admission to aneurysm repair, evidence of rebleeding). vi) Treatment specifications (EVT or clip surgery, along with details of the method used). vii) Procedure-related complications (thrombo-emboli, vessel occlusion, aneurysm rupture/perforation).

The prospective outcome information retrieved from the database included: i) The primary outcome variable was mortality, automatically updated from the National Population registry (Folkeregisteret; including the date of death), with the last update for all included patients completed by December 31 st, 2023. ii) Secondary outcome variables were assessed approximately six months after aneurysm repair and encompassed the modified Rankin Score (mRS), Glasgow Outcome Score Extended (GOSE), radiological evidence of brain infarction, duration of hospital stays, the use of tracheostomy, procedural-related complications, and the use of hemicraniectomy.

### Statistical analysis

Continuous variables were presented as either mean ± standard deviation with *P*-values derived from a two-sample t-test, or as median ± interquartile range (IQR) with *P*-values from a Kruskal–Wallis test, depending on distribution. Categorical data were presented as frequency with percentages, and *P*-values were calculated using a Pearson Chi-square test. Mortality (or survival) after surgery was determined with the Kaplan–Meier survival function, and the equality of survival functions across groups was assessed using the log-rank test. In the case of no event (i.e., death), patients were censored on 31 st December 2023 for all time-to-event (survival) analyses. No patients were lost to follow-up. The difference in the Kaplan–Meier survival function between groups at specific time points was assessed by a z-test, calculated using the respective standard errors from each survivor function. Both univariable and multivariable Cox proportional hazards models (Cox regression) were conducted and reported with hazard ratios (HR) and 95% confidence intervals (CI). Two-side *p*-values < 0.05 were considered significant.

## Results

### Patient cohort

From a total cohort of 1,402 patients, 205 individuals were excluded from the analysis for the following reasons: i) Aneurysm repair carried out at our institution on patients from other countries or from other regions within our country, thereby hindering follow-up (*n* = 31). ii) Aneurysm repair conducted at other institutions with patients subsequently transferred to our institution for intensive care management (*n* = 19). iii) Patients where no aneurysm repair occurred, either due to the inability to identify cerebral aneurysms (*n* = 14) or because extremely poor clinical conditions did not warrant aneurysm repair (*n* = 128). iv) Patients wrongly listed in the patient registry with no SAH (*n* = 13). Among the remaining 1,197 patients, 26 (2.1%) underwent aneurysm repair through a combination of surgery and EVT; these 26 individuals were not included in the analysis.

The remaining 1,171 patients encompassed all aSAH patients from this institution's catchment area that underwent clip surgery or EVT during the 12-year period 2011—2022 (Table 1).

**Table 1:** Pre-treatment information of total patient cohort, surgery group and EVT group

	Total	Surgery	EVT	*P*-value
*N*	1,171 (100.0%)	573 (48.9%)	598 (51.1%)	
**Age (years)**	58.2 ± 13.9	57.6 ± 13.3	58.7 ± 14.4	0.168
**Sex**				
Female	762 (65.1%)	377 (65.8%)	385 (64.4%)	0.612
Male	409 (34.9%)	196 (34.2%)	213 (35.6%)	
**Arterial hypertension**				
No	772 (65.9%)	371 (64.7%)	401 (67.1%)	0.404
Yes	399 (34.1%)	202 (35.3%)	197 (32.9%)	
**Diabetes mellitus**				
No	1,111 (94.9%)	547 (95.5%)	564 (94.3%)	0.373
Yes	60 (5.1%)	26 (4.5%)	34 (5.7%)	
**Smoking**				
Never smoked	270 (28.7%)	144 (31.0%)	126 (26.5%)	0.167
Unknown	51 (5.4%)	26 (5.6%)	25 (5.3%)	
Previous smoker	126 (13.4%)	52 (11.2%)	74 (15.5%)	
Current smoker	494 (52.5%)	243 (52.3%)	251 (52.7%)	
**Hunt & Hess grade**				
1	247 (21.1%)	123 (21.5%)	124 (20.8%)	0.409
2	335 (28.6%)	151 (26.4%)	184 (30.8%)	
3	224 (19.1%)	112 (19.5%)	112 (18.8%)	
4	174 (14.9%)	94 (16.4%)	80 (13.4%)	
5	190 (16.2%)	93 (16.2%)	97 (16.2%)	
**Intracerebral hematoma (ICH)**				
None	811 (69.3%)	338 (59.1%)	473 (79.1%)	< 0.001
ICH < 2 cm	118 (10.1%)	62 (10.8%)	56 (9.4%)	
ICH 2–5 cm	143 (12.2%)	85 (14.9%)	58 (9.7%)	
ICH > 5 cm	98 (8.4%)	87 (15.2%)	11 (1.8%)	
**Modified Fisher**				
0	6 (0.5%)	4 (0.7%)	2 (0.3%)	0.722
1	395 (33.8%)	195 (34.1%)	200 (33.5%)	
2	25 (2.1%)	10 (1.7%)	15 (2.5%)	
3	544 (46.5%)	270 (47.2%)	274 (45.9%)	
4	199 (17.0%)	93 (16.3%)	106 (17.8%)	
**Le Roux score**				
Le Roux score < 8	971 (83.1%)	486 (85.0%)	485 (81.2%)	0.090
Le Roux score ≥ 8	198 (16.9%)	86 (15.0%)	112 (18.8%)	
**Aneurysm size (mm)**	6.0 [4.0—9.0]	6.0 [4.0—9.0]	6.0 [4.0—8.0]	0.891
**Acute subdural hematoma**				
No	1,101 (94.1%)	538 (93.9%)	563 (94.3%)	0.764
Yes	69 (5.9%)	35 (6.1%)	34 (5.7%)	
**Rebleed**				
No	1,055 (90.8%)	516 (90.7%)	539 (90.9%)	0.902
Yes	107 (9.2%)	53 (9.3%)	54 (9.1%)	
**Aneurysm location**				
ACOM	420 (35.9%)	166 (29.0%)	254 (42.5%)	< 0.001
BA	105 (9.0%)	9 (1.6%)	96 (16.1%)	
BBA-ICA	19 (1.6%)	8 (1.4%)	11 (1.8%)	
ICA	62 (5.3%)	21 (3.7%)	41 (6.9%)	
MCA	265 (22.6%)	260 (45.4%)	5 (0.8%)	
PCOM	177 (15.1%)	58 (10.1%)	119 (19.9%)	
Pericallosa	48 (4.1%)	19 (3.3%)	29 (4.8%)	
VA	75 (6.4%)	32 (5.6%)	43 (7.2%)	
**Hours from arrival to repair**	7.0 [2.5—12.9]	5.5 [1.1—12.6]	8.1 [3.2—13.0]	< 0.001
**Hours from ictus to repair**	14.9 [7.0—29.6]	13.8 [5.4—26.1]	15.9 [8.5—34.8]	< 0.001
**≥ 12 h from ictus to repair**				
No	467 (40.4%)	250 (44.1%)	217 (36.9%)	0.013
Yes	688 (59.6%)	317 (55.9%)	371 (63.1%)	

Continuous variables were presented as mean ± standard deviation with *P*-values derived from a two-sample t-test, or as median ± interquartile range (IQR) with *P*-values from a Kruskal–Wallis test. Categorical data were presented as frequency with percentages, and *P*-values were calculated using a Pearson Chi-square test

Clip surgery was carried out in 573 (48.9%) patients whereas 598 (51.1%) were managed through EVT. The surgical repair was obtained using clip ligature (*n* = 546), clip + bypass (*n* = 4), wrap repair (*n* = 4), or surgical therapeutic parent artery occlusion (*n* = 19). EVT was achieved through coil (*n* = 395), coil with balloon support (*n* = 136), coil + stent (*n* = 24), flow diverter, stent, or flow diverter + stent (*n* = 24), flow diverter + stent + coil (*n* = 4), glue (*n* = 1), woven endo bridge (WEB) (*n* = 3) and therapeutic parent artery occlusion (*n* = 11).

### Treatment results of total cohort of 1,171 patients

Table 1 gives an overview of information prior to aneurysm repair for the total cohort of 1,171 patients (65% women and 35% men). About 1/3 of them were in poor clinical grade: HH grade 4 in 14.9% and HH grade 5 in 16.2%. Aneurysm repair was obtained median 14.9 h [interquartile range (IQR) 7.0—29.6 h] after ictus.

There were no differences in age, sex, co-morbidity, smoking habits, preoperative Hunt & Hess grade and frequency of rebleeds between the two groups. Certain differences between the two treatment groups should be noted: larger size ICH (2–5 cm, or > 5 cm) were more common in the surgical group; this reflects our practice of preferring surgical repair in aSAH patients with space-occupying hematomas. Also reflecting on our practice, the treatment modality depended on the aneurysm location: most MCA aneurysms were allocated to clip surgery while most BA aneurysms underwent EVT.

Notably, EVT was performed significantly later than clip surgery, assessed either as time from arrival to aneurysm repair, time from ictus to repair or proportion of repair ≥ 12 h after ictus. Another important difference, in the surgery group, there was higher frequency of CSF drainage using EVD and/or LD. Additionally, tracheostomy as well as hemicraniectomy were performed more often in the surgery group (Table 3).

**Fig. 1:**
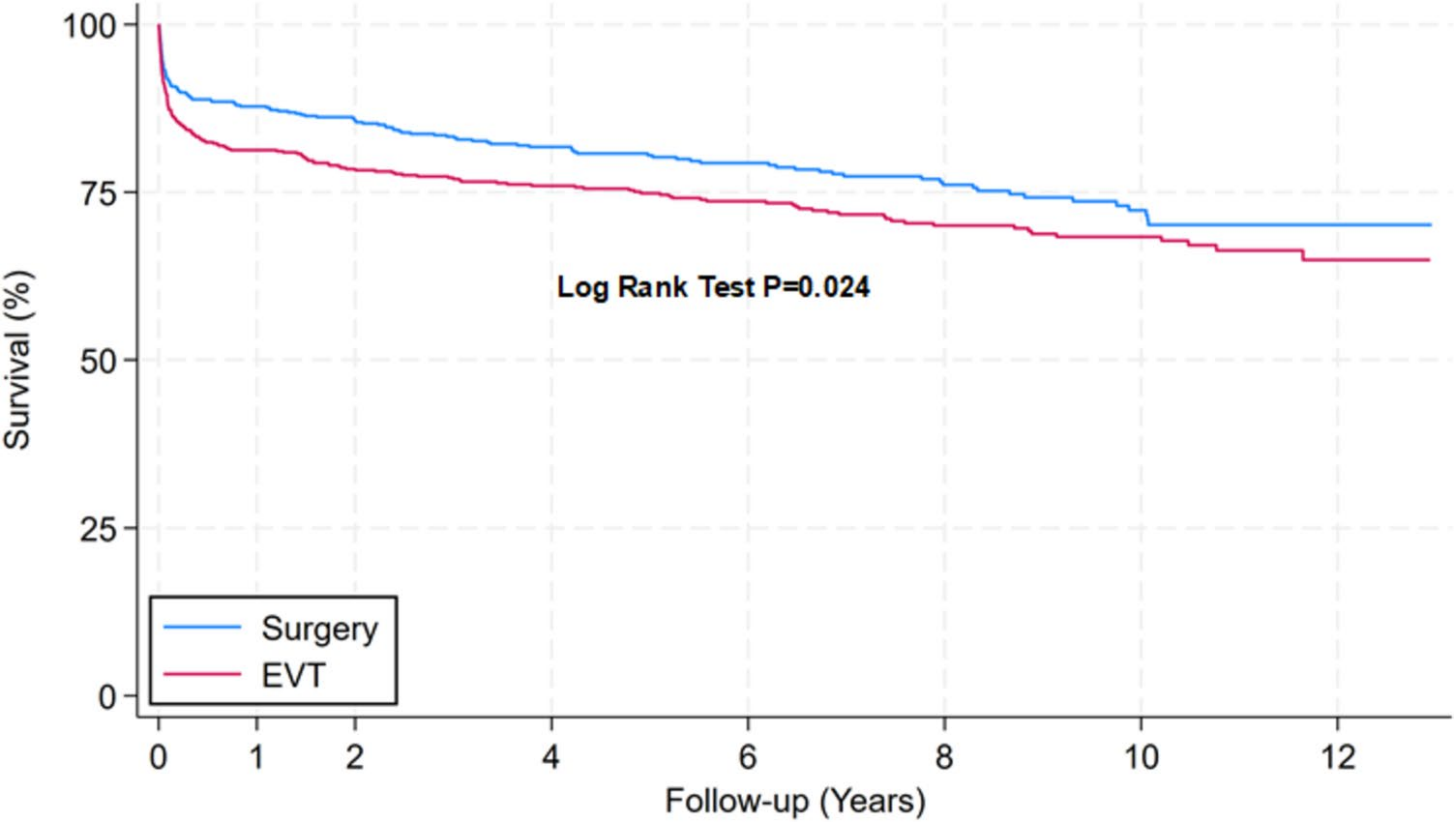
Survival curves of total material comparing mortality between patients undergoing surgery (blue line) or endovascular therapy (EVT, red line) for aneurysm repair after SAH. For total cohort of patients, Kaplan–Meier survival curves are shown for patients treated with either ETV (*n* = 598) or surgical (*n* = 573) modalities. Differences between survival curves were determined by Log Rank Test. Cox regression model showed a hazard ratio for death after EVT versus surgery (reference) of 1.302 (95%CI 1.036–1.637; *P* = 0.024). Patients at risk (with number dead in parenthesis during each follow-up interval): Year 0 [Surgery: 573 (71); EVT: 598 (112)]. Year 1 [Surgery: 502 (10); EVT: 486 (16)]. Year 2 [Surgery: 458 (20); EVT: 433 (13)]. Year 4 [Surgery: 343 (9); EVT: 363 (10)]. Year 6 [Surgery: 254 (9); EVT: 282 (12)]. Year 8 [Surgery: 180 (7); EVT: 198 (4)]. Year 10 [Surgery: 102 (3); EVT: 120 (4)]. Year 12 [Surgery: 35; EVT: 29]

Figure 1 reveals a lower mortality rate in the surgical than in the EVT group (*P* = 0.023; Log rank test). Table 2 presents the annual mortality rate, which was lower in the surgical group during the years one to eight. The 1-year, 5-year and 10-year mortality rate was 12.2%, 19.5% and 27.7% for the surgical group, while 18.7%, 25.2%, and 31.7% for the EVT group.

**Table 2:** Difference in mortality between surgery and EVT treatment groups from ictus to each year for all aneurysms, and ACOM and PCOM aneurysms

	All aneurysms			ACOM aneurysms			PCOM aneurysms		
Mortality	Surgery	EVT	Statistics	Surgery	EVT	Statistics	Surgery	EVT	Statistics
	*n* = 573	*n* = 598		*n* = 166	*n* = 254		*n* = 58	*n* = 119	
**Year 1**	12.4 (10.0–15.4)	18.7 (15.8–22.1)	**0.003**	10.2 (6.5–16.0)	16.5 (12.5–21.7)	0.057	10.3 (4.8–21.6)	16.0 (10.5–23.9)	0.282
**Year 2**	14.2 (11.6–17.3)	21.5 (18.4–25.1)	**0.001**	12.8 (8.5–18.9)	18.6 (14.3–24.0)	0.105	10.3 (4.8–21.6)	20.7 (14.4–29.3)	0.060
**Year 3**	16.7 (13.9–20.1)	23.0 (19.8–26.7)	**0.007**	13.4 (9.1–19.7)	19.9 (15.4–25.3)	0.080	16.2 (8.8–29.0)	22.9 (16.1–31.8)	0.298
**Year 4**	18.3 (15.3–21.8)	24.1 (20.8–27.7)	**0.017**	14.2 (9.7–20.7)	21.2 (16.6–26.8)	0.066	16.2 (8.8–29.0)	24.0 (17.0–33.1)	0.229
**Year 5**	19.5 (16.4–23.1)	25.2 (21.8–28.9)	**0.023**	15.2 (10.4–21.9)	22.2 (17.5–28.0)	0.072	16.2 (8.8–29.0)	25.2 (18.0–34.5)	0.170
**Year 6**	20.6 (17.4–24.4)	26.4 (22.9–30.2)	**0.026**	15.2 (10.4–21.9)	23.9 (19.0–29.8)	**0.029**	16.2 (8.8–29.0)	26.5 (19.1–36.1)	0.118
**Year 7**	22.6 (19.1–26.7)	28.3 (24.7–32.4)	**0.037**	19.0 (13.2–27.0)	26.4 (21.1–32.7)	0.107	19.2 (10.7–33.3)	28.4 (20.5–38.5)	0.208
**Year 8**	23.9 (20.2–28.1)	30.0 (26.2–34.2)	**0.034**	19.0 (13.2–27.0)	28.6 (23.0–35.2)	**0.041**	19.2 (10.7–33.3)	32.5 (23.5–43.7)	0.083
**Year 9**	25.8 (21.8–30.4)	31.2 (27.2–35.6)	0.073	19.0 (13.2–27.0)	29.5 (23.7–36.3)	**0.027**	19.2 (10.7–33.3)	35.2 (25.4–47.3)	**0.045**
**Year 10**	27.7 (23.3–32.7)	31.7 (27.6–36.1)	0.216	21.5 (14.5–31.0)	30.4 (24.5–37.4)	0.093	19.2 (10.7–33.3)	35.2 (25.4–47.3)	**0.045**
**Year 11**	29.9 (25.1–35.4)	33.7 (29.2–38.6)	0.276	24.2 (16.2–35.2)	34.5 (27.5–42.7)	0.095	19.2 (10.7–33.3)	35.2 (25.4–47.3)	**0.045**
**Year 12**	29.9 (25.1–35.4)	35.2 (30.1–40.8)	0.163	24.2 (16.2–35.2)	37.6 (29.0–47.7)	**0.048**	19.2 (10.7–33.3)	35.2 (25.4–47.3)	** < 0.001**

Data presented as mean probability (95%CI in parenthesis) estimated from a Kaplan–Meier survival function. Annual statistical difference determined by log Rank test, a z-test based on the respective standard errors. Ns: Non-significant

The patients stayed at our institution for 15.6 ± 8.4 days, with no difference between the groups (Table 3). The clinical outcome for the total patient cohort at average 6.9 months was good (mRS 0–2) in 70.4%, dependent (mRS 3–5) in 15.2% while 14.4% of the patients had died (Table 3). Both the modified Rankin Score (mRS) and the Glasgow Outcome Score Extended (GOSE) was significantly poorer in the EVT group as compared to the surgical group (Table 3). In contrast, the surgical cases showed more often radiologically documented brain infarction and underwent more frequently cerebrospinal fluid (CSF) drainage, tracheotomy and hemicraniectomy.

**Table 3:** Outcome for total patient cohort, surgery group and EVT group

	Total	Surgery	EVT	*P*-value
**N**	1,171 (100.0%)	573 (48.9%)	598 (51.1%)	
**Months after ictus**	6.9 ± 5.0	7.1 ± 4.8	6.8 ± 5.2	0.313
**Modified Rankin Score (mRS)**				
0	166 (14.3%)	77 (13.6%)	89 (15.0%)	0.001
1	441 (38.0%)	209 (36.9%)	232 (39.1%)	
2	210 (18.1%)	109 (19.3%)	101 (17.0%)	
3	63 (5.4%)	40 (7.1%)	23 (3.9%)	
4	86 (7.4%)	50 (8.8%)	36 (6.1%)	
5	27 (2.3%)	18 (3.2%)	9 (1.5%)	
6	167 (14.4%)	63 (11.1%)	104 (17.5%)	
**Glasgow Outcome Score Extended**				
1	167 (14.4%)	63 (11.1%)	104 (17.5%)	0.009
2	8 (0.7%)	4 (0.7%)	4 (0.7%)	
3	44 (3.8%)	29 (5.1%)	15 (2.5%)	
4	60 (5.2%)	36 (6.4%)	24 (4.0%)	
5	66 (5.7%)	37 (6.5%)	29 (4.9%)	
6	185 (15.9%)	95 (16.8%)	90 (15.2%)	
7	430 (37.1%)	209 (36.9%)	221 (37.2%)	
8	200 (17.2%)	93 (16.4%)	107 (18.0%)	
**Radiological brain infarction**				
Absent	624 (53.5%)	254 (44.4%)	370 (62.2%)	< 0.001
Present	543 (46.5%)	318 (55.6%)	225 (37.8%)	
**Days of hospital stay**	15.6 ± 8.4	15.8 ± 8.2	15.3 ± 8.5	0.323
**CSF drainage (EVD and/or LD)**				
No	222 (19.0%)	87 (15.2%)	135 (22.6%)	0.001
Yes	949 (81.0%)	486 (84.8%)	463 (77.4%)	
**Tracheotomy**				
No	840 (71.7%)	395 (68.9%)	445 (74.4%)	0.037
Yes	331 (28.3%)	178 (31.1%)	153 (25.6%)	
**Hemicraniectomy**				
No	1,140 (97.4%)	551 (96.2%)	589 (98.5%)	0.013
Yes	31 (2.6%)	22 (3.8%)	9 (1.5%)	
**Procedure-related complications**				
No	801 (68.4%)	381 (66.5%)	420 (70.2%)	0.169
Yes	370 (31.6%)	192 (33.5%)	178 (29.8%)	

Continuous variables were presented as mean ± standard deviation with *P*-values derived from a two-sample t-test. Categorical data were presented as frequency with percentages, and *P*-values were calculated using a Pearson Chi-square test. EVD: External ventricular drainage; CSF: Cerebrospinal fluid; LD: Lumbar drainage

Using different models, we analyzed possible explanations for the higher mortality rate in the EVT as compared to the surgical group. The hazard ratio for death for EVT versus clip surgery was 1.302 (95% CI 1.036 to 1.637; *P* = 0.024) from the univariable (unadjusted) Cox regression analysis. Considering the pretreatment variables using multivariable (adjusted) Cox regression analysis, the hazard ratio for EVT versus clip surgery increased to 1.443 (95% CI 1.054 to 1.976; *P* = 0.022; Table 4).

**Table 4:** Multivariable (adjusted) Cox regression analysis for total material

Factors	Hazard ratio	95%CI	*P*-value
**Treatment [EVT vs Surgery (= Reference)]**	1.443	1.054—1.976	0.022
**Age (years)**	1.058	1.046—1.070	< 0.001
**Sex (Male = Reference)**	0.671	0.521–0.864	0.002
**Arterial hypertension**	1.183	0.922–1.519	0.186
**Diabetes mellitus**	1.875	1.226–2.867	0.004
**Hunt & Hess grade**	1.512	1.344–1.702	< 0.001
**Intracerebral hematoma (ICH)**	1.049	0.912–1.207	0.500
**Modified Fisher**	1.055	0.911–1.222	0.471
**Le Roux** [< 8 (= Reference)/≥ 8]	1.512	1.080–2.101	0.014
**Acute subdural hematoma**	1.302	0.855–1.982	0.218
**Rebleed**	1.259	0.889–1.783	0.194
**Aneurysm groups (ACOM = Reference)**			
Pericallosa	0.839	0.427–1.650	0.611
MCA	1.266	0.847–1.893	0.249
ICA	0.612	0.320–1.170	0.138
PCOM	0.862	0.590–1.260	0.444
BBA-ICA	0.661	0.159–2.752	0.569
BA	1.090	0.712–1.669	0.692
VA	0.809	0.500–1.308	0.387
**Hours from arrival to repair**	1.004	0.999–1.008	0.111
**Hours from ictus to repair**	0.998	0.996–1.001	0.297
**≥ 12 h from ictus to repair**	1.156	0.881–1.517	0.295
**Procedure-related complications**	1.313	1.017–1.697	0.037

### Treatment results of ACOM and PCOM aneurysms

**Fig. 2:**
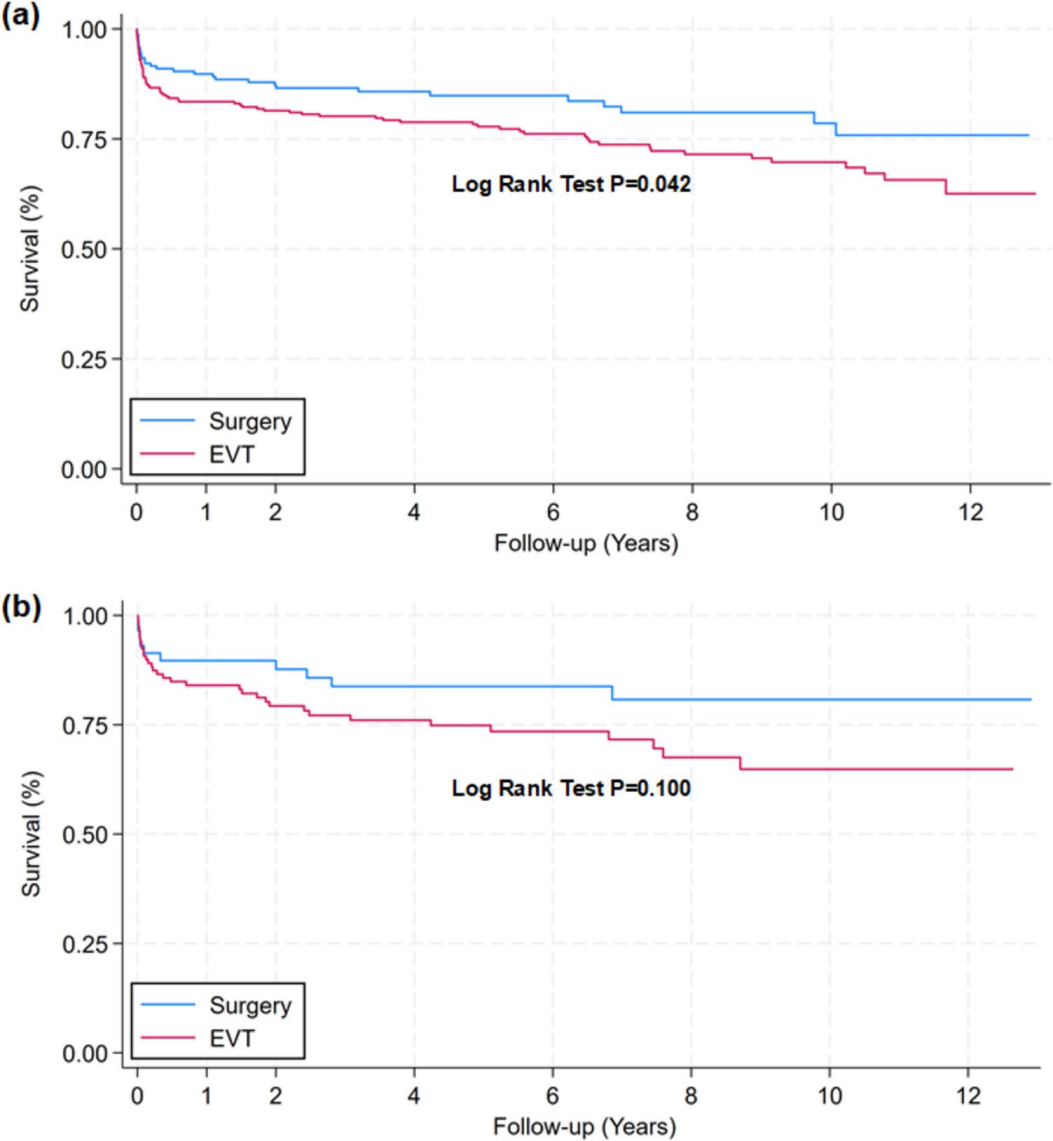
Survival curves of individuals with SAH from ACOM or PCOM aneurysms comparing mortality between patients undergoing endovascular or surgical repair of either ACOM or PCOM aneurysms after SAH**.** Kaplan–Meier survival curves for patients treated with either ETV or surgical modalities for (**a**) A1/ACOM aneurysms (EVT n = 254; surgery n = 166) or (**b**) PCOM aneurysms (EVT n = 119; surgery n = 58). Differences between survival curves were determined by Log Rank Test. Cox regression model showed a hazard ratio for death after EVT versus surgery (reference) for (**a**) ACOM aneurysms of 1.564 (95%CI 1.015–2.410; *P* = 0.043), and for (**b**) PCOM aneurysms of 1.798 (95%CI 0.885–3.652; *P* = 0.105). (**a**) Patients at risk (with number dead during each follow-up interval in parenthesis) for A1/ACOM aneurysms: Year 0 [Surgery: 166 (17); EVT: 254 (42)]. Year 1 [Surgery: 149 (4); EVT: 212 (5)]. Year 2 [Surgery: 133 (2); EVT: 196 (6)]. Year 4 [Surgery: 95 (1); EVT: 170 (5)]. Year 6 [Surgery: 71 (3); EVT: 131 (7)]. Year 8 [Surgery: 51 (1); EVT: 91 (2)]. Year 10 [Surgery: 29 (1); EVT: 58 (4)]. Year 12 [Surgery: 10; EVT: 15]. (b) Patients at risk (with number dead during each follow-up interval in parenthesis) for PCOM aneurysms: Year 0 [Surgery: 58 (6); EVT: 119 (19)]. Year 1 [Surgery: 52 (0); EVT: 100 (5)]. Year 2 [Surgery: 46 (3); EVT: 82 (3)]. Year 4 [Surgery: 36 (0); EVT: 65 (2)]. Year 6 [Surgery: 29 (1); EVT: 46 (3)]. Year 8 [Surgery: 22 (0); EVT: 31 (1)]. Year 10 [Surgery: 13 (0); EVT: 18 (0)]. Year 12 [Surgery: 1; EVT: 3]

We next compared outcome of clip surgery and EVT in patients with bleeds from either ACOM or PCOM aneurysms as these cohorts contained the highest numbers of each treatment modality. The pre-treatment information for each group is presented in Table 5. Kaplan–Meier curves for survival showed a lower mortality rate in the surgical than in the EVT group for ACOM (Log rank test *P* = 0.041; Fig. 2a) but not for PCOM aneurysms (Log rank test *P* = 0.100; Fig. 2b). The mortality rate was, however, lower in the surgical group after some years for both the ACOM and PCOM aneurysms (Table 2). For the ACOM aneurysms, 1-year, 5-year and 10-year mortality rate was 10.2%, 15.2% and 21.5% for clip surgery, while 16.5%, 22.2%, and 30.4% for the EVT group. Conversely, for the PCOM aneurysms, 1-year, 5-year and 10-year mortality was 10.3%, 16.2% and 19.2% for the surgical group, while 16.0%, 25.1%, and 35.2% for the EVT group.

**Table 5:** Pre-treatment information about patients with ACOM or PCOM aneurysms

	ACOM			PCOM		
	Surgery	EVT	*P*-value	Surgery	EVT	*P*-value
**N**	166 (39.5%)	254 (60.5%)		58 (32.8%)	119 (67.2%)	
**Age (years)**	55.8 ± 12.8	57.9 ± 14.4	0.131	59.9 ± 15.0	63.8 ± 13.9	0.085
**Sex**						
Female	89 (53.6%)	136 (53.5%)	0.989	39 (67.2%)	90 (75.6%)	0.239
Male	77 (46.4%)	118 (46.5%)		19 (32.8%)	29 (24.4%)	
**Arterial hypertension**						
No	105 (63.3%)	178 (70.1%)	0.145	38 (65.5%)	69 (58.0%)	0.336
Yes	61 (36.7%)	76 (29.9%)		20 (34.5%)	50 (42.0%)	
**Diabetes mellitus**						
No	157 (94.6%)	248 (97.6%)	0.099	55 (94.8%)	105 (88.2%)	0.162
Yes	9 (5.4%)	6 (2.4%)		3 (5.2%)	14 (11.8%)	
**Smoking**						
Never smoked	47 (33.6%)	51 (26.6%)	0.331	15 (31.2%)	25 (23.6%)	0.515
Unknown	6 (4.3%)	12 (6.2%)		1 (2.1%)	3 (2.8%)	
Previous smoker	17 (12.1%)	18 (9.4%)		5 (10.4%)	20 (18.9%)	
Current smoker	70 (50.0%)	111 (57.8%)		27 (56.2%)	58 (54.7%)	
**Hunt & Hess**						
1	40 (24.1%)	59 (23.3%)	0.631	15 (25.9%)	23 (19.3%)	0.284
2	56 (33.7%)	85 (33.6%)		23 (39.7%)	48 (40.3%)	
3	31 (18.7%)	36 (14.2%)		6 (10.3%)	24 (20.2%)	
4	19 (11.4%)	32 (12.6%)		7 (12.1%)	17 (14.3%)	
5	20 (12.0%)	41 (16.2%)		7 (12.1%)	7 (5.9%)	
**Intracerebral hematoma (ICH)**						
None	112 (67.5%)	176 (69.3%)	0.098	43 (74.1%)	103 (86.6%)	0.065
ICH < 2 cm	27 (16.3%)	33 (13.0%)		5 (8.6%)	10 (8.4%)	
ICH 2–5 cm	17 (10.2%)	39 (15.4%)		6 (10.3%)	4 (3.4%)	
ICH > 5 cm	10 (6.0%)	6 (2.4%)		4 (6.9%)	2 (1.7%)	
**Modified Fisher**						
0	1 (0.6%)	1 (0.4%)	0.577	0 (0.0%)	1 (0.8%)	0.676
1	53 (32.1%)	95 (37.4%)		23 (39.7%)	42 (35.3%)	
2	2 (1.2%)	7 (2.8%)		0 (0.0%)	1 (0.8%)	
3	79 (47.9%)	113 (44.5%)		27 (46.6%)	64 (53.8%)	
4	30 (18.2%)	38 (15.0%)		8 (13.8%)	11 (9.2%)	
**Le Roux score**						
Le Roux score < 8	138 (83.6%)	215 (84.6%)	0.782	51 (87.9%)	105 (88.2%)	0.953
Le Roux score ≥ 8	27 (16.4%)	39 (15.4%)		7 (12.1%)	14 (11.8%)	
**Aneurysm size (mm)**	6.0 [4.0—8.0]	6.0 [4.0—8.0]	0.515	8.0 [5.0—10.0]	6.0 [5.0—8.0]	0.289
**Acute subdural hematoma**						
No	158 (95.2%)	242 (95.3%)	0.964	52 (89.7%)	110 (92.4%)	0.533
Yes	8 (4.8%)	12 (4.7%)		6 (10.3%)	9 (7.6%)	
**Rebleed**						
No	153 (92.7%)	232 (91.7%)	0.703	55 (94.8%)	114 (95.8%)	0.770
Yes	12 (7.3%)	21 (8.3%)		3 (5.2%)	5 (4.2%)	
**Hours from arrival to repair**	7.8 [2.0—14.0]	7.2 [3.0—12.3]	0.740	7.4 [1.3—12.1]	8.0 [3.1—12.9]	0.240
**Hours from ictus to repair**	15.4 [7.3—26.2]	16.6 [8.4—47.8]	0.135	14.7 [8.5—22.3]	16.7 [9.3—52.3]	0.108
**≥ 12 h from ictus to repair**						
No	63 (38.2%)	88 (35.5%)	0.577	21 (36.2%)	41 (34.5%)	0.818
Yes	102 (61.8%)	160 (64.5%)		37 (63.8%)	78 (65.5%)	

Continuous variables were presented as mean ± standard deviation with *P*-values derived from a two-sample t-test, or as median ± interquartile range (IQR) with *P*-values from a Kruskal–Wallis test. Categorical data were presented as frequency with percentages, and *P*-values were calculated using a Pearson Chi-square test

Outcome at about 7 months for the ACOM and PCOM aneurysm locations are presented in Table 6. No significant differences in mRS score were found between EVT and surgical groups. As we further found no differences between the mRS and GOSE outcome scores, we present only the mRS scores in Table 6. Notably, CSF drainage was more frequent in the surgery group.

**Table 6:** Outcome of patients with ACOM or PCOM aneurysms

	ACOM			PCOM		
	Surgery	EVT	*P*-value	Surgery	EVT	*P*-value
**N**	166 (39.5%)	254 (60.5%)		58 (32.8%)	119 (67.2%)	
**Months after ictus**	7.1 ± 5.0	7.4 ± 5.8	0.557	7.7 ± 5.4	6.3 ± 4.5	0.077
**Modified Rankin Score (mRS)**						
0	23 (14.0%)	41 (16.2%)	0.498	9 (15.8%)	20 (16.8%)	0.585
1	69 (42.1%)	103 (40.7%)		20 (35.1%)	46 (38.7%)	
2	28 (17.1%)	39 (15.4%)		12 (21.1%)	22 (18.5%)	
3	10 (6.1%)	11 (4.3%)		4 (7.0%)	2 (1.7%)	
4	16 (9.8%)	16 (6.3%)		4 (7.0%)	9 (7.6%)	
5	3 (1.8%)	5 (2.0%)		2 (3.5%)	2 (1.7%)	
6	15 (9.1%)	38 (15.0%)		6 (10.5%)	18 (15.1%)	
**Radiological brain infarction**						
Absent	78 (47.0%)	149 (59.1%)	0.015	32 (55.2%)	86 (72.3%)	0.024
Present	88 (53.0%)	103 (40.9%)		26 (44.8%)	33 (27.7%)	
**Days of hospital stay**	16.9 ± 7.5	14.7 ± 7.5	0.005	14.5 ± 8.1	15.6 ± 9.4	0.481
**CSF drainage (EVD and/or LD)**						
No	12 (7.2%)	63 (24.8%)	< 0.001	14 (24.1%)	30 (25.2%)	0.877
Yes	154 (92.8%)	191 (75.2%)		44 (75.9%)	89 (74.8%)	
**Tracheotomy**						
No	123 (74.1%)	188 (74.0%)	0.985	45 (77.6%)	97 (81.5%)	0.538
Yes	43 (25.9%)	66 (26.0%)		13 (22.4%)	22 (18.5%)	
**Hemicraniectomy**						
No	164 (98.8%)	251 (98.8%)	0.983	56 (96.6%)	118 (99.2%)	0.207
Yes	2 (1.2%)	3 (1.2%)		2 (3.4%)	1 (0.8%)	
**Procedure-related complications**						
No	103 (62.0%)	174 (68.5%)	0.172	40 (69.0%)	93 (78.2%)	0.184
Yes	63 (38.0%)	80 (31.5%)		18 (31.0%)	26 (21.8%)	

Continuous variables were presented as mean ± standard deviation with P-values derived from a two-sample t-test. Categorical data were presented as frequency with percentages, and P-values were calculated using a Pearson Chi-square test

When specifically addressing the ACOM aneurysms, univariable (unadjusted) Cox regression showed a hazard ratio for EVT versus clip surgery of 1.564 (95% CI 1.015 to 2.410; *P* = 0.043). Considering the pretreatment variables in multivariable (adjusted) Cox regression analysis, the hazard ratio for EVT versus surgery was 1.570 (95% CI 0.976 to 2.525; *P* = 0.063; Table 7). For the PCOM aneurysms, the univariable (unadjusted) Cox regression showed a hazard ratio for EVT versus surgery of 1.798 (95% CI 0.885 to 3.652; *P* = 0.105). Considering the pretreatment variables in multivariable (adjusted) Cox regression analysis, hazard ratio for EVT versus clip surgery was 1,326 (95% CI 0.579 to 3.037; *P* = 0.504; Table 7).

**Table 7:** Multivariable (adjusted) Cox regression analysis addressing ACOM and PCOM aneurysms

	ACOM aneurysms			PCOM aneurysms		
Factors	Hazard ratio	95%CI	*P*-value	Hazard ratio	95%CI	*P*-value
**Treatment [EVT vs Surgery (= Reference)]**	1.570	0.976–2.525	0.063	1.326	0.579–3.037	0.504
**Age (years)**	1.049	1.028–1.071	< 0.001	1.070	1.037–1.105	< 0.001
**Sex (Male = Reference)**	0.834	0.527–1.318	0.437	0.513	0.241–1.091	0.083
**Arterial hypertension**	1.605	1.035–2.490	0.035	1.211	0.593–2.473	0.600
**Diabetes mellitus**	4.087	1.937–8.624	< 0.001	1.269	0.501–3.215	0.616
**Hunt & Hess grade**	1.769	1.431–2.186	< 0.001	1.248	0.876–1.778	0.220
**Intracerebral hematoma (ICH)**	1.015	0.793–1.300	0.904	0.840	0.506–1.397	0.502
**Modified Fisher**	1.185	0.903–1.555	0.221	1.308	0.848–2.017	0.224
**Le Roux** [< 8 (= Reference)/> 8]	1.467	0.792–2.717	0.223	1.166	0.389–3.496	0.784
**Acute subdural hematoma**	1.675	0.799–3.492	0.173	1.902	0.565–6.399	0.299
**Rebleed**	2.081	1.157–3.743	0.014	0.829	0.187–3.672	0.805
**Hours from arrival to repair**	1.006	0.991–1.021	0.436	1.010	0.983–1.038	0.483
**Hours from ictus to repair**	1.000	0.995 −1.005	0.992	1.001	0.995–1.007	0.771
**≥ 12 h from ictus to repair**	0.988	0.611–1.596	0.959	1.173	0.522–2.637	0.700
**Procedure-related complications**	1.997	1.294–3.081	0.002	0.707	0.299–1.668	0.428

The distribution of clip surgery and EVT remained stable throughout the study period, as illustrated in Supplementary Fig. 1.

### Treatment results of other specific aneurysm locations

**Fig. 3:**
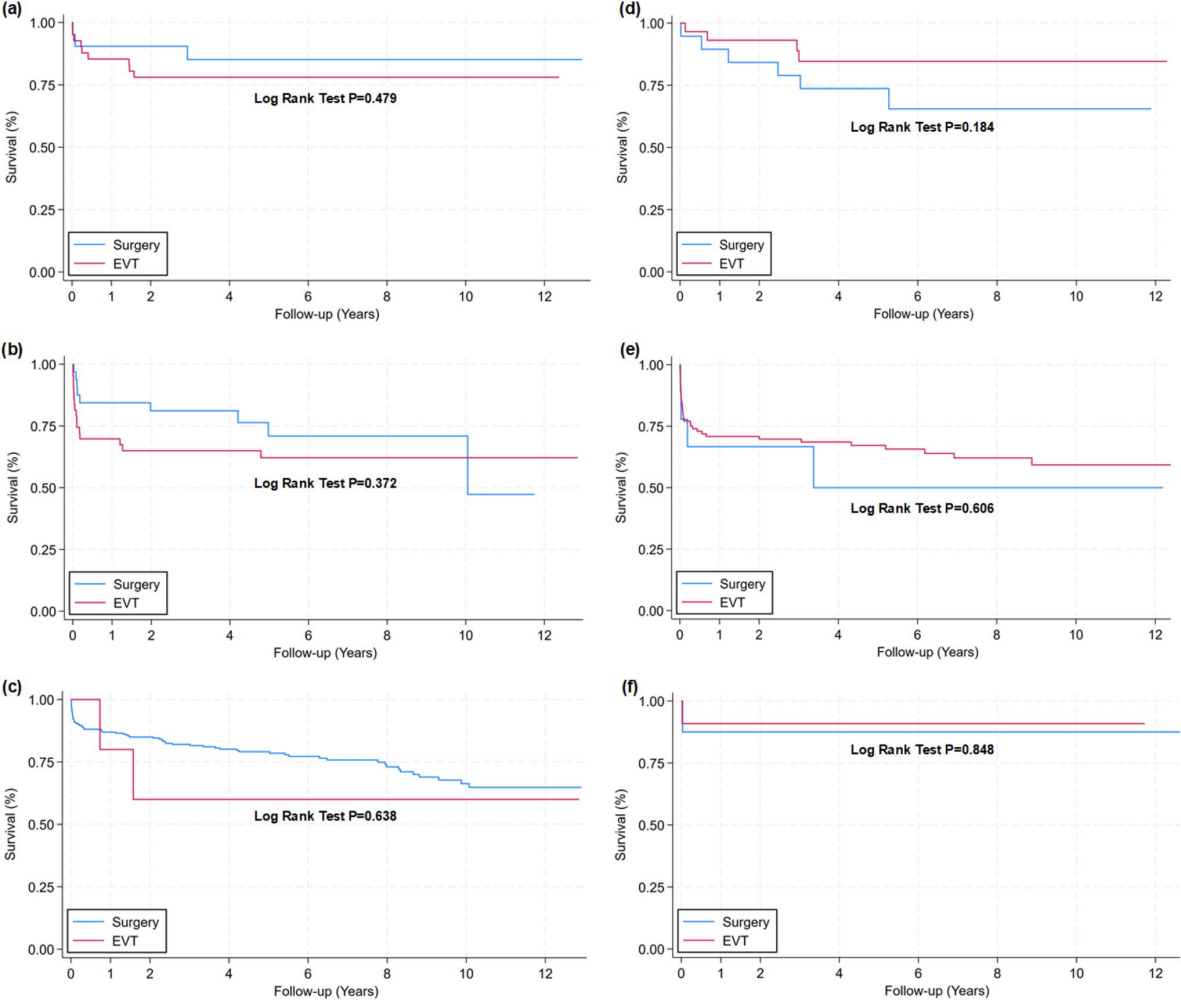
Survival curves for defined aneurysm categories. Comparison of outcome between patients undergoing endovascular (EVT) or surgical aneurysm repair after SAH for different aneurysm categories. Kaplan–Meier survival curves for patients treated with either ETV or surgery for the aneurysm categories (**a**) ICA (EVT n = 41; Surg n = 21), (**b**) VA (EVT n = 43; Surg n = 32), (**c**) MCA (EVT n = 5; Surg n = 260), (**d**) pericallosal artery (EVT n = 29; Surg n = 19), (**e**) BA (EVT n = 96; Surg n = 9) and (**f**) BBA-ICA (EVT n = 11; Surg n = 8). Differences between survival curves were determined by Log Rank Test. Cox regression model showed a hazard ratio for death after EVT versus surgery for aneurysm categories (**a**) ICA of 1.596 (95%CI 0.432–5.896; *P* = 0.483), (**b**) VA of 1.449 (95%CI 0.638–3.289; *P* = 0.376), (**c**) MCA of 1.404 (95%CI 0.344–5.738; *P* = 0.637), (**d**) pericallosal artery aneurysms of 0.435 (95%CI 0.122–1.542; *P* = 0.197), (**e**) BA aneurysms (BA top/mid, and SCA confluence) of 0.762 (95%CI 0.271–2.147; *P* = 0.607), and (f) BBA-ICA aneurysms of 0.763 (95%CI 0.048–12.204; *P* = 0.848). (**a**) Patients at risk (with number dead during each follow-up interval in parenthesis) for ICA aneurysms: Year 0 [Surgery: 21 (2); EVT: 41 (6)]. Year 1 [Surgery: 19 (0); EVT: 35 (3)]. Year 2 [Surgery: 19 (1); EVT: 32 (0)]. Year 4 [Surgery: 15 (0); EVT: 28 (0)]. Year 6 [Surgery: 11 (0); EVT: 25 (0)]. Year 8 [Surgery: 9 (0); EVT: 21 (0)]. Year 10 [Surgery: 5 (0); EVT: 8 (0)]. Year 12 [Surgery: 3; EVT: 2]. (**b**) Patients at risk (with number dead during each follow-up interval in parenthesis) for VA aneurysms: Year 0 [Surgery: 32 (5); EVT: 43 (13)]. Year 1 [Surgery: 27 (1); EVT: 30 (2)]. Year 2 [Surgery: 25 (0); EVT: 25 (0)]. Year 4 [Surgery: 18 (2); EVT: 23 (1)]. Year 6 [Surgery: 13 (0); EVT: 18 (0)]. Year 8 [Surgery: 9 (0); EVT: 13 (0)]. Year 10 [Surgery: 3 (1); EVT: 9 (0)]. Year 12 [Surgery: 0; EVT: 2]. (**c**) Patients at risk (with number dead during each follow-up interval in parenthesis) for MCA aneurysms: Year 0 [Surgery: 260 (34); EVT: 5 (1)]. Year 1 [Surgery: 226 (5); EVT: 4 (1)]. Year 2 [Surgery: 207 (11); EVT: 3 (0)]. Year 4 [Surgery: 156 (5); EVT: 3 (0)]. Year 6 [Surgery: 114 (5); EVT: 3 (0)]. Year 8 [Surgery: 78 (6); EVT: 2 (0)]. Year 10 [Surgery: 45 (1); EVT: 2 (0)]. Year 12 [Surgery: 19; EVT: 1]. (**d**) Patients at risk (with number dead during each follow-up interval in parenthesis) for pericallosal aneurysms: Year 0 [Surgery: 19 (2); EVT: 29 (2)]. Year 1 [Surgery: 17 (1); EVT: 27 (0)]. Year 2 [Surgery: 16 (2); EVT: 23 (2)]. Year 4 [Surgery: 14 (1); EVT: 18 (0)]. Year 6 [Surgery: 7 (0); EVT: 17 (0)]. Year 8 [Surgery: 4 (0); EVT: 13 (0)]. Year 10 [Surgery: 2 (0); EVT: 10 (0)]. Year 12 [Surgery: 0; EVT: 2]. (**e**) Patients at risk (with number dead during each follow-up interval in parenthesis) for BA aneurysms: Year 0 [Surgery: 9 (3); EVT: 96 (28)]. Year 1 [Surgery: 6 (0); EVT: 68 (0)]. Year 2 [Surgery: 5 (1); EVT: 65 (2)]. Year 4 [Surgery: 3 (0); EVT: 53 (2)]. Year 6 [Surgery: 3 (0); EVT: 40 (2)]. Year 8 [Surgery: 2 (0); EVT: 26 (1)]. Year 10 [Surgery: 1 (0); EVT: 14 (0)]. Year 12 [Surgery: 1; EVT: 4]. (**f**) Patients at risk (with number dead during each follow-up interval in parenthesis) for BBA-ICA aneurysms: Year 0 [Surgery: 8 (1); EVT: 11 (1)]. Year 1 [Surgery: 7 (0); EVT: 10 (0)]. Year 2 [Surgery: 7 (0); EVT: 7 (0)]. Year 4 [Surgery: 6 (0); EVT: 3 (0)]. Year 6 [Surgery: 6 (0); EVT: 2 (0)]. Year 8 [Surgery: 5 (0); EVT: 1 (0)]. Year 10 [Surgery: 4 (0); EVT: 1 (0)]. Year 12 [Surgery: 2; EVT: 0]

Figure 3 shows survival curves for the aneurysm locations ICA (Fig. 3a), VA (Fig. 3b), MCA (Fig. 3c), pericallosal artery (Fig. 3d), BA (Fig. 3e) and BBA-ICA (Fig. 3f). No significant differences in survival between the two groups were found.

Pre-treatment information and outcome results of clip surgery versus EVT groups is provided for the specific aneurysm locations in the Supplementary Material, as follows: ICA aneurysms (S-Tables 1–2), VA aneurysms (S-Table 3–4), MCA aneurysms (S-Tables 5–6), pericallosal artery aneurysms (S-Tables 7–8), BA aneurysms (S-Tables 9–10) and BBA-ICA aneurysms (S-Tables 11–12). In general, the pre-treatment and outcome data showed lack of significant differences between the two treatment groups. Some differences may, however, be noted. For the VA aneurysms, despite significantly worse HH grade in the surgery group (S-Table 3) at admission, outcome at about half a year (S-Table 4) was not different between groups. Furthermore, for BBA-ICA aneurysms, although the pre-treatment HH grade was significantly worse in the EVT group (S-Table 11), there was no difference in outcome at about 7 months (S-Table 12). For more details about our experience with treating BBA-ICA aneurysms, see [5].

## Discussion

The main finding from this study, which included all our aSAH patients treated either surgically or with EVT and for whom data were collected prospectively, is a significantly higher mortality rate in patients treated with EVT compared to those who underwent clip surgery. This disparity persisted both across the overall patient cohort after statistically adjusting for patient characteristics and with the same tendency within the subgroup specifically comprising patients with ACOM and PCOM aneurysms. Plausible explanations are the maintenance of surgical skills and prompt reduction of intracranial pressure (ICP).

A strength of this study is the prospective data collection, meaning that data were gathered at the time of patient treatment or follow-up according to a predefined protocol. This design enhances data completeness and minimizes bias, offering the advantages associated with prospective studies, even though the analysis was done retrospectively.

Mortality was selected as the primary outcome of this study, given its reliability as a clear and indisputable endpoint. Within the framework of the current study, there were no missing data on mortality, as information on all included patients was available through the National Population Registry (Folkeregisteret), last updated on December 31 st, 2023.

Among our patients, 31.1% were classified as Hunt Hess poor grade (4–5) prior to aneurysm repair. For the total cohort, the 1-year, 5-year, and 10-year mortality rates were 12.4%, 19.5%, and 27.7% for clip surgery, and 18.7%, 25.2%, and 31.7% for the EVT group. These figures compare favorably with previous studies reporting mortality rates in surgery versus EVT [16, 22]. For example, in the ISAT trial consisting almost exclusively of Hunt & Hess good grade patients, the mortality at one year was 8.1% among 801 EVT treated and 10.1% in the 793 patients allocated to surgery [16].

What might be plausible explanations for the higher mortality rate in our EVT group? Regarding the pretreatment variables, the EVT group presented with a longer duration from ictus to aneurysm repair (see Table 1). The time from ictus to aneurysm repair is a significant factor in poor grade aSAH patients, contributing to the worsened outcomes [21]. Also, in the ISAT study, differences in favor of EVT were linked to a longer time from ictus to aneurysm repair in the surgical group [23]. Therefore, longer time to treatment in the EVT group could possibly contribute to the higher mortality. Conversely, the incidence of intracerebral hemorrhage (ICH) was presently higher in the surgical group, serving as a negative predictor for outcomes. No differences were observed regarding the clinical state before aneurysm repair, as indicated by the percentage of Hunt & Hess grade.

The differences in mortality cannot be attributed to shifts in treatment modality over time, as the distribution of EVT and clip surgery remained stable throughout the study period. Perioperative management was also the same for the two patient groups and carried out by the same therapists [20, 21].

Searching other possible reasons for higher mortality in the EVT group, one consistent difference between the groups is the faster reduction of ICP in the surgical group. This is due to the shorter time to treatment, the use of craniotomy for blood removal, and more rapid and extensive drainage of cerebrospinal fluid (CSF). The surgical group had a higher rate of CSF drainage using EVD and/or LD as well a higher rate of hemicraniectomy. While the latter may partly reflect a greater incidence of intracerebral hematoma and radiological evidence of brain infarction, hemicraniectomy itself can significantly improve ICP control. Furthermore, CSF drainage is an effective way to obtain control of ICP and intracranial compliance (i.e., intracranial pressure–volume relationship), which we measure according to the mean ICP wave amplitude (MWA) [4, 7]. It is reasonable to speculate that earlier and more effective ICP management, i.e., keeping ICP low and well beyond the threshold of 20 mmHg, contributes to the lower mortality observed in the surgical group. Supporting this, we previously demonstrated in a randomized controlled trial that improving the intracranial compliance through MWA-guided intensive care (largely obtained by CSF drainage) significantly improved outcomes, as measured by the modified Rankin Scale at 12 months [6]. Surveillance according to MWA led to lower ICP scores. In that study, 12-month mortality was 14.6% in the MWA-guided group compared to 18.4% in the group managed according to traditional ICP thresholds. Therefore, earlier, more frequent and thus higher CSF volume drainage leads to lower ICP that may explain the lower mortality in the surgical group.

Regarding secondary outcome, as assessed by both the mRS and GOSE scales, we presently identified a poorer outcome in the EVT group for the total patient cohort. This difference was, however, not observed for individual aneurysm locations. The observation time compared between the groups. Notably, mRS and GOSE were assessed approximately half a year after aneurysm repair and represents outcome measures that are more open to interpretation than mortality, the primary and more unequivocal endpoint.

It is crucial to note that the mRS and GOSE scales present only a general and broad indication of the outcome. Aneurysmal SAH patients may thus exhibit favorable mRS and GOSE scores while still experiencing complaints such as fatigue, cognitive impairment, and headaches, collectively giving rise to work-related challenges [10, 24]. These latter complaints were not evaluated in the present study.

Following the ISAT study, there has been a substantial shift globally towards adopting the endovascular treatment approach, largely replacing surgical aneurysm repair [1, 19]. With an equal proportion undergoing clip surgery and EVT, our institution diverges from this trend and positions us advantageously for comparing the efficacy of the two treatment modalities.

Based on the presently reported experience, we cannot assert that EVT is superior to clip surgery for aneurysm repair in aSAH. On the contrary, our mortality rates were higher in the EVT group, albeit potentially attributable to factors such as longer duration before aneurysm repair. This compares with observations in poor grad aneurysmal SAH [25].

In numerous institutions, endovascular modalities have predominantly supplanted clip surgery. This shift, however, comes with the consequence of diminishing neurosurgical expertise. The present results do not endorse a such change; instead, our results suggest the necessity of preserving the surgical skills for effective aneurysm repair. Maintaining microsurgical expertise for aneurysmal repair in aSAH is hence mandatory.

Several limitations in the present study should be acknowledged. Firstly, patients were not randomized to either EVT or clip surgery. Therefore, systematic differences between the groups cannot be excluded, though the patient cohorts allocated to each treatment modality were, in many respects, comparable. Moreover, significance levels were maintained after adjusting for differences in variables between groups.

Another limitation in comparing the two cohorts was the distribution of aneurysm locations between the two groups. Comparable results were, however, identified for the total cohort as well as for the subgroups of ACOM or PCOM aneurysm locations, which had the highest patient numbers in each group. Comparisons of treatment modalities for the other aneurysm locations were impeded by the low patient numbers in each treatment group. It may also be considered a limitation that we did not include data on aneurysm morphology, though aneurysm size was included in the analysis. Finally, although we have no missing data regarding date of death, the exact causes of death were unknown, which may be considered a limitation, especially with regards to possible explanations to higher mortality in the EVT group.

## Conclusion

The present study revealed a higher mortality rate in the aSAH patient cohort undergoing EVT as compared to those managed by clip surgery. This trend was observed across the entire cohort as well as in the patient subgroups treated for ruptured ACOM or PCOM aneurysms. This underscores the importance of ongoing surveillance of treatment outcomes, ideally guiding the selection of the most appropriate treatment modality. Maintaining surgical skills for aneurysm repair in aSAH is mandatory. A plausible explanation for the lower mortality in the surgery group is the earlier, more frequent and thus more extensive CSF drainage leading to lower ICP that optimizes the intracranial compliance (i.e., improved intracranial pressure–volume relationship).

## Supplementary Information

Below is the link to the electronic supplementary material.

Supplementary file 1 (PDF 894 KB)

## Data Availability

No datasets were generated or analysed during the current study.

## Abbreviations

ACA: Anterior cerebral artery
ACOM: Anterior communicating artery
BA: Basilar artery
BBA: Blood-blister aneurysm
CI: Confidence interval
EVT: Endovascular therapy
GOSE: Glasgow Outcome Score Extended
HH: Hunt-Hess
ICA: Internal carotid artery
IQR: Interquartile range
MCA: Middle cerebral artery
mRS: Modified Rankin score
PCOM: Posterior communicating artery
SAH: Subarachnoid hemorrhage
SCA: Superior cerebellar artery
SURG: Surgery
VA: Vertebral artery
