# Supplementary material for: Comparative analysis of surgical and endovascular aneurysm repair in subarachnoid hemorrhage: a single-center study with 1,171 patients
Source: Acta Neurochir (Wien). 2025 Sep 13;167(1):244. doi: 10.1007/s00701-025-06670-w (PMC12433440; doi:10.1007/s00701-025-06670-w)
Supplement: Supplementary file 1 — (PDF 894 KB) [file 701_2025_6670_MOESM1_ESM.pdf]

Supplementary Material

Comparative Analysis of Surgical and Endovascular Aneurysm Repair in Subarachnoid Hemorrhage: A Single-Center Experience with 1,171 Patients

Per Kristian Eide[1,2,3,*] • Wilhelm Sorteberg[1] • Are H. Pripp[3,4,5] • Pål A. Rønning[1] • Angelika G Sorteberg[1,2]

[1]Department of Neurosurgery, Oslo University Hospital - Rikshospitalet, Oslo, Norway
[2]Institute of Clinical Medicine, Faculty of Medicine, University of Oslo, Oslo, Norway
[3]KG Jebsen Centre for Brain Fluid Research, University of Oslo, Oslo, Norway
[4]Oslo Centre of Biostatistics and Epidemiology, Research Support Services, Oslo University Hospital, Oslo, Norway.
[5]Faculty of Health Sciences, Oslo Metropolitan University, Oslo, Norway.

Correspondence to:
Professor Per Kristian Eide, MD PhD
Department of Neurosurgery
Oslo University Hospital - Rikshospitalet
Pb 4950 Nydalen,
Phone: +47 91649419
Fax: +47-23074310
N-0424 Oslo, Norway
p.k.eide@medisin.uio.no

Supplementary Figure 1. Distribution of EVT versus surgical clipping during the study period

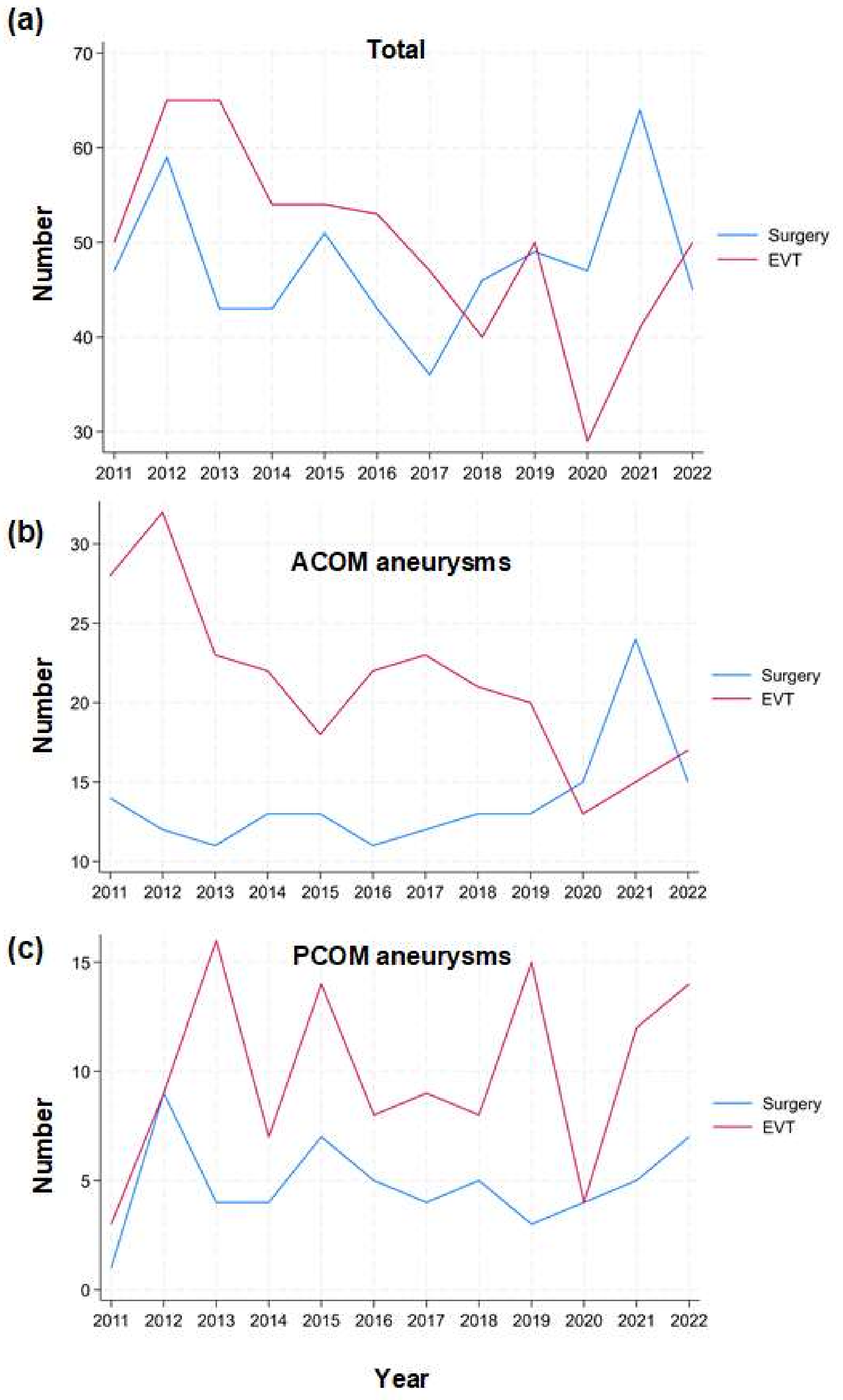

Change over time in number of patients undergoing endovascular (EVT, red line) or surgical (Surgery, blue line) aneurysm repair after SAH for different aneurysm categories. (a) Total material, **(b)** ACOM aneurysms, and **(c)** PCOM aneurysms.

Supplementary Table 1. Pre-treatment information about patients with ICA aneurysms

	Total	Surgery	EVT	P-value
**N**	62 (100.0%)	21 (33.9%)	41 (66.1%)	
**Age (years)**	55.2 ± 13.4	52.9 ± 14.5	56.3 ± 12.8	0.339
**Sex**				
Female	49 (79.0%)	14 (66.7%)	35 (85.4%)	0.087
Male	13 (21.0%)	7 (33.3%)	6 (14.6%)	
**Arterial hypertension**				
No	48 (77.4%)	17 (81.0%)	31 (75.6%)	0.634
Yes	14 (22.6%)	4 (19.0%)	10 (24.4%)	
**Diabetes mellitus**				
No	56 (90.3%)	18 (85.7%)	38 (92.7%)	0.380
Yes	6 (9.7%)	3 (14.3%)	3 (7.3%)	
**Smoking**				
Never smoked	17 (34.0%)	5 (33.3%)	12 (34.3%)	0.995
Unknown	4 (8.0%)	1 (6.7%)	3 (8.6%)	
Previous smoker	3 (6.0%)	1 (6.7%)	2 (5.7%)	
Current smoker	26 (52.0%)	8 (53.3%)	18 (51.4%)	
**Hunt & Hess**				
1	11 (17.7%)	3 (14.3%)	8 (19.5%)	0.897
2	17 (27.4%)	5 (23.8%)	12 (29.3%)	
3	12 (19.4%)	5 (23.8%)	7 (17.1%)	
4	10 (16.1%)	3 (14.3%)	7 (17.1%)	
5	12 (19.4%)	5 (23.8%)	7 (17.1%)	
**Intracerebral hematoma (ICH)**				
None	47 (75.8%)	16 (76.2%)	31 (75.6%)	0.425
ICH <2 cm	6 (9.7%)	1 (4.8%)	5 (12.2%)	
ICH 2-5 cm	8 (12.9%)	3 (14.3%)	5 (12.2%)	
ICH >5 cm	1 (1.6%)	1 (4.8%)	0 (0.0%)	
**Modified Fisher**				
1	23 (37.1%)	6 (28.6%)	17 (41.5%)	0.555
3	25 (40.3%)	9 (42.9%)	16 (39.0%)	
4	14 (22.6%)	6 (28.6%)	8 (19.5%)	
**Le Roux score**				
Le Roux score < 8	49 (79.0%)	17 (81.0%)	32 (78.0%)	0.790
Le Roux score ≥ 8	13 (21.0%)	4 (19.0%)	9 (22.0%)	
**Aneurysm size (mm)**	5.5 [4.0 - 14.0]	5.0 [3.0 - 15.0]	7.0 [4.0 - 13.0]	0.587
**Acute subdural hematoma**				
No	56 (90.3%)	19 (90.5%)	37 (90.2%)	0.977
Yes	6 (9.7%)	2 (9.5%)	4 (9.8%)	
**Rebleed**				
No	53 (86.9%)	17 (81.0%)	36 (90.0%)	0.320
Yes	8 (13.1%)	4 (19.0%)	4 (10.0%)	
**Aneurysm Group**				
ICA	62 (100.0%)	21 (100.0%)	41 (100.0%)	.
**Hours from arrival to repair**	8.0 [2.6 - 13.0]	12.5 [1.0 - 17.0]	7.7 [3.5 - 11.1]	0.380
**Hours from ictus to repair**	12.9 [7.7 - 24.0]	17.0 [9.5 - 36.1]	12.5 [7.7 - 21.4]	0.422
**>12 hours from ictus to repair**				

No	27 (43.5%)	8 (38.1%)	19 (46.3%)	0.535
Yes	35 (56.5%)	13 (61.9%)	22 (53.7%)	

Continuous variables were presented as mean ± standard deviation with P-values derived from a two-sample t-test, or as median ± interquartile range (IQR) with P-values from a Kruskal-Wallis test. Categorical data were presented as frequency with percentages, and P-values were calculated using a Pearson Chi-square test.

Supplementary Table 2. Outcome of patients with ICA aneurysms

	Total	Surgery	EVT	P-value
**N**	62 (100.0%)	21 (33.9%)	41 (66.1%)	
**Months after ictus**	6.7 ± 4.8	7.6 ± 5.4	6.3 ± 4.4	0.300
**Modified Rankin Score (mRS)**				
0	7 (11.5%)	2 (9.5%)	5 (12.5%)	0.245
1	24 (39.3%)	5 (23.8%)	19 (47.5%)	
2	13 (21.3%)	7 (33.3%)	6 (15.0%)	
3	5 (8.2%)	3 (14.3%)	2 (5.0%)	
4	3 (4.9%)	2 (9.5%)	1 (2.5%)	
5	1 (1.6%)	0 (0.0%)	1 (2.5%)	
6	8 (13.1%)	2 (9.5%)	6 (15.0%)	
**Radiological brain infarction**				
Absent	42 (67.7%)	13 (61.9%)	29 (70.7%)	0.482
Present	20 (32.3%)	8 (38.1%)	12 (29.3%)	
**Days of hospital stay**	15.3 ± 8.3	17.2 ± 8.5	14.4 ± 8.1	0.207
**CSF drainage (EVD and/or LD)**				
No	16 (25.8%)	3 (14.3%)	13 (31.7%)	0.138
Yes	46 (74.2%)	18 (85.7%)	28 (68.3%)	
**Tracheotomy**				
No	45 (72.6%)	14 (66.7%)	31 (75.6%)	0.455
Yes	17 (27.4%)	7 (33.3%)	10 (24.4%)	
**Hemicraniectomy**				
No	59 (95.2%)	20 (95.2%)	39 (95.1%)	0.984
Yes	3 (4.8%)	1 (4.8%)	2 (4.9%)	
**Procedure-related complications**				
No	51 (82.3%)	16 (76.2%)	35 (85.4%)	0.371
Yes	11 (17.7%)	5 (23.8%)	6 (14.6%)	

Continuous variables were presented as mean ± standard deviation with P-values derived from a two-sample t-test, or as median ± interquartile range (IQR) with P-values from a Kruskal-Wallis test. Categorical data were presented as frequency with percentages, and P-values were calculated using a Pearson Chi-square test.

Supplementary Table 3. Pre-treatment information about patients with VA aneurysms

	Total	Surgery	EVT	P-value
**N**	75 (100.0%)	32 (42.7%)	43 (57.3%)	
**Age (years)**	62.0 ± 13.6	62.3 ± 11.8	61.7 ± 14.9	0.842
**Sex**				
Female	54 (72.0%)	24 (75.0%)	30 (69.8%)	0.618
Male	21 (28.0%)	8 (25.0%)	13 (30.2%)	
**Arterial hypertension**				
No	49 (65.3%)	20 (62.5%)	29 (67.4%)	0.656
Yes	26 (34.7%)	12 (37.5%)	14 (32.6%)	
**Diabetes mellitus**				
No	69 (92.0%)	30 (93.8%)	39 (90.7%)	0.630
Yes	6 (8.0%)	2 (6.2%)	4 (9.3%)	
**Smoking**				
Never smoked	24 (41.4%)	12 (44.4%)	12 (38.7%)	0.770
Unknown	5 (8.6%)	3 (11.1%)	2 (6.5%)	
Previous smoker	9 (15.5%)	3 (11.1%)	6 (19.4%)	
Current smoker	20 (34.5%)	9 (33.3%)	11 (35.5%)	
**Hunt & Hess**				
1	11 (14.7%)	4 (12.5%)	7 (16.3%)	0.048
2	16 (21.3%)	4 (12.5%)	12 (27.9%)	
3	14 (18.7%)	9 (28.1%)	5 (11.6%)	
4	5 (6.7%)	0 (0.0%)	5 (11.6%)	
5	29 (38.7%)	15 (46.9%)	14 (32.6%)	
**Intracerebral hematoma (ICH)**				
None	61 (81.3%)	22 (68.8%)	39 (90.7%)	0.030
ICH <2 cm	7 (9.3%)	4 (12.5%)	3 (7.0%)	
ICH 2-5 cm	7 (9.3%)	6 (18.8%)	1 (2.3%)	
**Modified Fisher**				
1	18 (24.0%)	6 (18.8%)	12 (27.9%)	0.432
2	5 (6.7%)	1 (3.1%)	4 (9.3%)	
3	19 (25.3%)	8 (25.0%)	11 (25.6%)	
4	33 (44.0%)	17 (53.1%)	16 (37.2%)	
**Le Roux score**				
Le Roux score < 8	40 (53.3%)	15 (46.9%)	25 (58.1%)	0.333
Le Roux score ≥ 8	35 (46.7%)	17 (53.1%)	18 (41.9%)	
**Aneurysm size (mm)**	5.0 [3.0 - 6.0]	4.0 [3.0 - 5.0]	5.0 [4.0 - 6.5]	0.073
**Acute subdural hematoma**				
No	68 (90.7%)	29 (90.6%)	39 (90.7%)	0.991
Yes	7 (9.3%)	3 (9.4%)	4 (9.3%)	
**Rebleed**				
No	64 (85.3%)	27 (84.4%)	37 (86.0%)	0.840
Yes	11 (14.7%)	5 (15.6%)	6 (14.0%)	
**Aneurysm Group**				
VA	75 (100.0%)	32 (100.0%)	43 (100.0%)	.
**Hours from arrival to repair**	6.8 [2.7 - 14.5]	5.4 [2.5 - 15.2]	9.0 [3.2 - 14.5]	0.463
**Hours from ictus to repair**	13.7 [6.0 - 22.4]	10.0 [4.5 - 21.8]	14.1 [7.7 - 33.5]	0.156
**≥12 hours from ictus to repair**				

No	34 (45.3%)	17 (53.1%)	17 (39.5%)	0.242
Yes	41 (54.7%)	15 (46.9%)	26 (60.5%)	

Continuous variables were presented as mean ± standard deviation with P-values derived from a two-sample t-test, or as median ± interquartile range (IQR) with P-values from a Kruskal-Wallis test. Categorical data were presented as frequency with percentages, and P-values were calculated using a Pearson Chi-square test.

Supplementary Table 4. Outcome of patients with VA aneurysms

	Total	Surgery	EVT	P-value
**N**	75 (100.0%)	32 (42.7%)	43 (57.3%)	
**Months after ictus**	5.6 ± 4.0	6.1 ± 3.4	5.3 ± 4.3	0.369
**Modified Rankin Score (mRS)**				
0	10 (13.5%)	3 (9.4%)	7 (16.7%)	0.112
1	24 (32.4%)	10 (31.2%)	14 (33.3%)	
2	15 (20.3%)	10 (31.2%)	5 (11.9%)	
3	4 (5.4%)	1 (3.1%)	3 (7.1%)	
4	2 (2.7%)	2 (6.2%)	0 (0.0%)	
5	1 (1.4%)	1 (3.1%)	0 (0.0%)	
6	18 (24.3%)	5 (15.6%)	13 (31.0%)	
**Radiological brain infarction**				
Absent	42 (56.0%)	18 (56.2%)	24 (55.8%)	0.970
Present	33 (44.0%)	14 (43.8%)	19 (44.2%)	
**Days of hospital stay**	16.4 ± 7.8	17.9 ± 8.4	15.2 ± 7.2	0.142
**CSF drainage (EVD and/or LD)**				
No	13 (17.3%)	4 (12.5%)	9 (20.9%)	0.340
Yes	62 (82.7%)	28 (87.5%)	34 (79.1%)	
**Tracheotomy**				
No	43 (57.3%)	13 (40.6%)	30 (69.8%)	0.012
Yes	32 (42.7%)	19 (59.4%)	13 (30.2%)	
**Hemicraniectomy**				
No	74 (98.7%)	32 (100.0%)	42 (97.7%)	0.385
Yes	1 (1.3%)	0 (0.0%)	1 (2.3%)	
**Procedure-related complications**				
No	52 (69.3%)	25 (78.1%)	27 (62.8%)	0.154
Yes	23 (30.7%)	7 (21.9%)	16 (37.2%)	

Continuous variables were presented as mean ± standard deviation with P-values derived from a two-sample t-test, or as median ± interquartile range (IQR) with P-values from a Kruskal-Wallis test. Categorical data were presented as frequency with percentages, and P-values were calculated using a Pearson Chi-square test.

Supplementary Table 5. Pre-treatment information about patients with MCA aneurysms

	Total	Surgery	EVT	P-value
**N**	265 (100.0%)	260 (98.1%)	5 (1.9%)	
**Age (years)**	58.1 ± 13.1	58.2 ± 13.0	54.6 ± 15.9	0.546
**Sex**				
Female	188 (70.9%)	186 (71.5%)	2 (40.0%)	0.124
Male	77 (29.1%)	74 (28.5%)	3 (60.0%)	
**Arterial hypertension**				
No	168 (63.4%)	163 (62.7%)	5 (100.0%)	0.086
Yes	97 (36.6%)	97 (37.3%)	0 (0.0%)	
**Diabetes mellitus**				
No	257 (97.0%)	252 (96.9%)	5 (100.0%)	0.690
Yes	8 (3.0%)	8 (3.1%)	0 (0.0%)	
**Smoking**				
Never smoked	59 (28.2%)	59 (28.4%)	0 (0.0%)	0.690
Unknown	13 (6.2%)	13 (6.2%)	0 (0.0%)	
Previous smoker	22 (10.5%)	22 (10.6%)	0 (0.0%)	
Current smoker	115 (55.0%)	114 (54.8%)	1 (100.0%)	
**Hunt & Hess**				
1	53 (20.0%)	52 (20.0%)	1 (20.0%)	0.758
2	58 (21.9%)	56 (21.5%)	2 (40.0%)	
3	59 (22.3%)	58 (22.3%)	1 (20.0%)	
4	54 (20.4%)	54 (20.8%)	0 (0.0%)	
5	41 (15.5%)	40 (15.4%)	1 (20.0%)	
**Intracerebral hematoma (ICH)**				
None	129 (48.9%)	125 (48.3%)	4 (80.0%)	0.432
ICH <2 cm	22 (8.3%)	22 (8.5%)	0 (0.0%)	
ICH 2-5 cm	46 (17.4%)	45 (17.4%)	1 (20.0%)	
ICH >5 cm	67 (25.4%)	67 (25.9%)	0 (0.0%)	
**Modified Fisher**				
0	2 (0.8%)	2 (0.8%)	0 (0.0%)	0.744
1	98 (37.0%)	97 (37.3%)	1 (20.0%)	
2	7 (2.6%)	7 (2.7%)	0 (0.0%)	
3	133 (50.2%)	129 (49.6%)	4 (80.0%)	
4	25 (9.4%)	25 (9.6%)	0 (0.0%)	
**Le Roux score**				
Le Roux score < 8	241 (90.9%)	236 (90.8%)	5 (100.0%)	0.476
Le Roux score ≥ 8	24 (9.1%)	24 (9.2%)	0 (0.0%)	
**Aneurysm size (mm)**	7.0 [4.0 - 10.0]	7.0 [4.0 - 10.0]	7.0 [4.0 - 9.0]	0.974
**Acute subdural hematoma**				
No	251 (94.7%)	246 (94.6%)	5 (100.0%)	0.594
Yes	14 (5.3%)	14 (5.4%)	0 (0.0%)	
**Rebleed**				
No	239 (91.2%)	235 (91.4%)	4 (80.0%)	0.371
Yes	23 (8.8%)	22 (8.6%)	1 (20.0%)	
**Aneurysm group**				
MCA	265 (100.0%)	260 (100.0%)	5 (100.0%)	.
**Hours from arrival to repair**	3.1 [1.0 - 10.4]	3.1 [0.9 - 10.4]	9.5 [4.2 - 10.0]	0.153

**Hours from ictus to repair**	12.2 [4.7 - 29.6]	12.2 [4.6 - 28.1]	12.5 [8.2 - 135.5]	0.320
**≥12 hours from ictus to repair**				
No	128 (49.2%)	126 (49.4%)	2 (40.0%)	0.677
Yes	132 (50.8%)	129 (50.6%)	3 (60.0%)	

Continuous variables were presented as mean ± standard deviation with P-values derived from a two-sample t-test, or as median ± interquartile range (IQR) with P-values from a Kruskal-Wallis test. Categorical data were presented as frequency with percentages, and P-values were calculated using a Pearson Chi-square test.

Supplementary Table 6. Outcome of patients with MCA aneurysms

	Total	Surgery	EVT	P-value
**N**	265 (100.0%)	260 (98.1%)	5 (1.9%)	
**Months after ictus**	6.9 ± 4.7	6.9 ± 4.7	9.0 ± 4.8	0.324
**Modified Rankin Score (mRS)**				
0	38 (14.6%)	37 (14.5%)	1 (20.0%)	0.929
1	97 (37.2%)	95 (37.1%)	2 (40.0%)	
2	45 (17.2%)	44 (17.2%)	1 (20.0%)	
3	20 (7.7%)	20 (7.8%)	0 (0.0%)	
4	24 (9.2%)	23 (9.0%)	1 (20.0%)	
5	7 (2.7%)	7 (2.7%)	0 (0.0%)	
6	30 (11.5%)	30 (11.7%)	0 (0.0%)	
**Radiological brain infarction**				
Absent	102 (38.6%)	99 (38.2%)	3 (60.0%)	0.322
Present	162 (61.4%)	160 (61.8%)	2 (40.0%)	
**Days of hospital stay**	14.7 ± 7.9	14.7 ± 7.9	16.0 ± 3.7)	0.713
**CSF drainage (EVD and/or LD)**				
No	49 (18.5%)	49 (18.8%)	0 (0%)	0.282
Yes	216 (81.5%)	211 (81.2%)	5 (100.0%)	
**Tracheotomy**				
No	181 (68.3%)	177 (68.1%)	4 (80.0%)	0.570
Yes	84 (31.7%)	83 (31.9%)	1 (20.0%)	
**Hemicraniectomy**				
No	250 (94.3%)	245 (94.2%)	5 (100.0%)	0.580
Yes	15 (5.7%)	15 (5.8%)	0 (0.0%)	
**Procedure-related complications**				
No	183 (69.1%)	179 (68.8%)	4 (80.0%)	0.593
Yes	82 (30.9%)	81 (31.2%)	1 (20.0%)	

Continuous variables were presented as mean ± standard deviation with P-values derived from a two-sample t-test, or as median ± interquartile range (IQR) with P-values from a Kruskal-Wallis test. Categorical data were presented as frequency with percentages, and P-values were calculated using a Pearson Chi-square test.

Supplementary Table 7. Pre-treatment information about patients with pericallosal aneurysms

	Total	Surgery	EVT	P-value
**N**	48 (100.0%)	19 (39.6%)	29 (60.4%)	
**Age**	56.3 ± 10.8)	56.7 ± 12.8	56.0 ± 9.5	0.811
**Sex**				
Female	34 (70.8%)	13 (68.4%)	21 (72.4%)	0.766
Male	14 (29.2%)	6 (31.6%)	8 (27.6%)	
**Arterial hypertension**				
No	38 (79.2%)	16 (84.2%)	22 (75.9%)	0.486
Yes	10 (20.8%)	3 (15.8%)	7 (24.1%)	
**Diabetes mellitus**				
No	48 (100.0%)	19 (100.0%)	29 (100.0%)	
Yes	0 (0%)	0 (0%)	0 (0%)	
**Smoking**				
Never smoked	8 (20.5%)	3 (20.0%)	5 (20.8%)	0.972
Unknown	2 (5.1%)	1 (6.7%)	1 (4.2%)	
Previous smoker	9 (23.1%)	3 (20.0%)	6 (25.0%)	
Current smoker	20 (51.3%)	8 (53.3%)	12 (50.0%)	
**Hunt & Hess**				
1	13 (27.1%)	5 (26.3%)	8 (27.6%)	0.076
2	10 (20.8%)	3 (15.8%)	7 (24.1%)	
3	7 (14.6%)	0 (0.0%)	7 (24.1%)	
4	10 (20.8%)	6 (31.6%)	4 (13.8%)	
5	8 (16.7%)	5 (26.3%)	3 (10.3%)	
**Intracerebral hematoma (ICH)**				
None	22 (45.8%)	5 (26.3%)	17 (58.6%)	0.140
ICH <2 cm	5 (10.4%)	2 (10.5%)	3 (10.3%)	
ICH 2-5 cm	13 (27.1%)	7 (36.8%)	6 (20.7%)	
ICH >5 cm	8 (16.7%)	5 (26.3%)	3 (10.3%)	
**Modified Fisher**				
1	18 (37.5%)	6 (31.6%)	12 (41.4%)	0.339
2	2 (4.2%)	0 (0.0%)	2 (6.9%)	
3	20 (41.7%)	8 (42.1%)	12 (41.4%)	
4	8 (16.7%)	5 (26.3%)	3 (10.3%)	
**Le Roux score**				
Le Roux score < 8	39 (81.2%)	14 (73.7%)	25 (86.2%)	0.277
Le Roux score ≥ 8	9 (18.8%)	5 (26.3%)	4 (13.8%)	
**Aneurysm size (mm)**	5.0 [4.0 - 7.0]	5.5 [4.0 - 7.0]	5.0 [4.0 - 7.0]	0.937
**Acute subdural hematoma**				
No	44 (91.7%)	17 (89.5%)	27 (93.1%)	0.656
Yes	4 (8.3%)	2 (10.5%)	2 (6.9%)	
**Rebleed**				
No	41 (85.4%)	16 (84.2%)	25 (86.2%)	0.848
Yes	7 (14.6%)	3 (15.8%)	4 (13.8%)	
**Aneurysm Group**				
Pericallosa	48 (100.0%)	19 (100.0%)	29 (100.0%)	.
**Hours from arrival to repair**	6.5 [2.5 - 11.00]	2.8 [0.9 10.6]	9.0 [3.1 11.6]	0.023
**Hours from ictus to repair**	13.0 [6.1 - 21.8]	7.0 [3.8 - 18.0]	15.5 [8.8 - 42.7]	0.016

**≥12 hours from ictus to repair**				
No	23 (48.9%)	11 (57.9%)	12 (42.9%)	0.312
Yes	24 (51.1%)	8 (42.1%)	16 (57.1%)	

Continuous variables were presented as mean ± standard deviation with P-values derived from a two-sample t-test, or as median ± interquartile range (IQR) with P-values from a Kruskal-Wallis test. Categorical data were presented as frequency with percentages, and P-values were calculated using a Pearson Chi-square test.

Supplementary Table 8. Outcome of patients with pericallosal aneurysms

	Total	Surgery	EVT	P-value
**N**	48 (100.0%)	19 (39.6%)	29 (60.4%)	
**Months after ictus**	7.2 ± 4.0	7.8 ± 4.2	6.8 ± 3.9	0.396
**Modified Rankin Score (mRS)**				
0	7 (14.6%)	2 (10.5%)	5 (17.2%)	0.071
1	16 (33.3%)	4 (21.1%)	12 (41.4%)	
2	13 (27.1%)	4 (21.1%)	9 (31.0%)	
3	2 (4.2%)	2 (10.5%)	0 (0.0%)	
4	4 (8.3%)	2 (10.5%)	2 (6.9%)	
5	4 (8.3%)	4 (21.1%)	0 (0.0%)	
6	2 (4.2%)	1 (5.3%)	1 (3.4%)	
**Radiological brain infarction**				
Absent	22 (45.8%)	6 (31.6%)	16 (55.2%)	0.109
Present	26 (54.2%)	13 (68.4%)	13 (44.8%)	
**Days of hospital stay**	16.8 ± 9.0	18.7 ± 9.2	15.5 ± 8.7	0.234
**CSF drainage (EVD and/or LD)**				
No	11 (22.9%)	1 (5.3%)	10 (34.5%)	0.019
Yes	37 (77.1%)	18 (94.7%)	19 (65.5%)	
**Tracheotomy**				
No	28 (58.3%)	10 (52.6%)	18 (62.1%)	0.517
Yes	20 (41.7%)	9 (47.4%)	11 (37.9%)	
**Hemicraniectomy**				
No	47 (97.9%)	19 (100.0%)	28 (96.6%)	0.413
Yes	1 (2.1%)	0 (0.0%)	1 (3.4%)	
**Procedure-related complications**				
No	29 (60.4%)	9 (47.4%)	20 (69.0%)	0.135
Yes	19 (39.6%)	10 (52.6%)	9 (31.0%)	

Continuous variables were presented as mean ± standard deviation with P-values derived from a two-sample t-test, or as median ± interquartile range (IQR) with P-values from a Kruskal-Wallis test. Categorical data were presented as frequency with percentages, and P-values were calculated using a Pearson Chi-square test.

Supplementary Table 9. Pre-treatment information about patients with BA aneurysms

	Total	Surgery	EVT	P-value
**N**	105 (100.0%)	9 (8.6%)	96 (91.4%)	
**Age (years)**	56.7 ± 15.6	64.3 ± 12.5	56.0 ± 15.7	0.127
**Sex**				
Female	73 (69.5%)	7 (77.8%)	66 (68.8%)	0.574
Male	32 (30.5%)	2 (22.2%)	30 (31.2%)	
**Arterial hypertension**				
No	68 (64.8%)	6 (66.7%)	62 (64.6%)	0.900
Yes	37 (35.2%)	3 (33.3%)	34 (35.4%)	
**Diabetes mellitus**				
No	99 (94.3%)	9 (100.0%)	90 (93.8%)	0.440
Yes	6 (5.7%)	0 (0.0%)	6 (6.2%)	
**Smoking**				
Never smoked	18 (21.4%)	2 (28.6%)	16 (20.8%)	0.701
Unknown	5 (6.0%)	1 (14.3%)	4 (5.2%)	
Previous smoker	20 (23.8%)	1 (14.3%)	19 (24.7%)	
Current smoker	41 (48.8%)	3 (42.9%)	38 (49.4%)	
**Hunt & Hess**				
1	21 (20.0%)	4 (44.4%)	17 (17.7%)	0.433
2	17 (16.2%)	1 (11.1%)	16 (16.7%)	
3	28 (26.7%)	2 (22.2%)	26 (27.1%)	
4	16 (15.2%)	1 (11.1%)	15 (15.6%)	
5	23 (21.9%)	1 (11.1%)	22 (22.9%)	
**Intracerebral hematoma (ICH)**				
None	103 (98.1%)	9 (100.0%)	94 (97.9%)	0.909
ICH <2 cm	1 (1.0%)	0 (0.0%)	1 (1.0%)	
ICH 2-5 cm	1 (1.0%)	0 (0.0%)	1 (1.0%)	
**Modified Fisher**				
0	1 (1.0%)	1 (11.1%)	0 (0.0%)	0.021
1	23 (22.1%)	2 (22.2%)	21 (22.1%)	
2	1 (1.0%)	0 (0.0%)	1 (1.1%)	
3	52 (50.0%)	5 (55.6%)	47 (49.5%)	
4	27 (26.0%)	1 (11.1%)	26 (27.4%)	
**Le Roux score**				
Le Roux score < 8	78 (75.0%)	8 (88.9%)	70 (73.7%)	0.314
Le Roux score ≥ 8	26 (25.0%)	1 (11.1%)	25 (26.3%)	
**Aneurysm size (mm)**	7.0 [4.0 - 12.0]	3.5 [3.0 - 5.0]	7.0 [4.0 - 12.0]	0.016
**Acute subdural hematoma**				
No	102 (98.1%)	9 (100.0%)	93 (97.9%)	0.660
Yes	2 (1.9%)	0 (0.0%)	2 (2.1%)	
**Rebleed**				
No	90 (87.4%)	8 (88.9%)	82 (87.2%)	0.886
Yes	13 (12.6%)	1 (11.1%)	12 (12.8%)	
**Aneurysm group**				
BA	105 (100.0%)	9 (100.0%)	96 (100.0%)	.
**Hours from arrival to repair**	9.5 [3.8 - 14.9]	16.0 [11.7 - 21.0]	8.6 [3.7 - 14.3]	0.094
**Hours from ictus to repair**	15.3 [8.2 - 28.0]	24.7 [15.2 - 72.5]	15.1 [8.2 - 23.9]	0.121

**≥12 hours from ictus to repair**				
No	39 (38.2%)	2 (22.2%)	37 (39.8%)	0.301
Yes	63 (61.8%)	7 (77.8%)	56 (60.2%)	

Continuous variables were presented as mean ± standard deviation with P-values derived from a two-sample t-test, or as median ± interquartile range (IQR) with P-values from a Kruskal-Wallis test. Categorical data were presented as frequency with percentages, and P-values were calculated using a Pearson Chi-square test.

Supplementary Table 10. Outcome of patients with BA aneurysms

	Total	Surgery	EVT	P-value
**N**	105 (100.0%)	9 (8.6%)	96 (91.4%)	
**Months after ictus**	6.3 ± 5.6	5.5 ± 4.8	6.4 ± 5.6	0.647
**Modified Rankin Score (mRS)**				
0	11 (10.6%)	1 (11.1%)	10 (10.5%)	0.833
1	33 (31.7%)	4 (44.4%)	29 (30.5%)	
2	17 (16.3%)	0 (0.0%)	17 (17.9%)	
3	4 (3.8%)	0 (0.0%)	4 (4.2%)	
4	8 (7.7%)	1 (11.1%)	7 (7.4%)	
5	1 (1.0%)	0 (0.0%)	1 (1.1%)	
6	30 (28.8%)	3 (33.3%)	27 (28.4%)	
**Radiological brain infarction**				
Absent	63 (60.6%)	6 (66.7%)	57 (60.0%)	0.696
Present	41 (39.4%)	3 (33.3%)	38 (40.0%)	
**Days of hospital stay**	15.8 ± 9.1	12.7 ± 6.5	16.1 ± 9.2	0.279
**CSF drainage (EVD and/or LD)**				
No	13 (12.4%)	3 (33.3%)	10 (10.4%)	0.046
Yes	92 (87.6%)	6 (66.7%)	86 (89.6%)	
**Tracheotomy**				
No	76 (72.4%)	8 (88.9%)	68 (70.8%)	0.247
Yes	29 (27.6%)	1 (11.1%)	28 (29.2%)	
**Hemicraniectomy**				
No	104 (99.0%)	9 (100.0%)	95 (99.0%)	0.758
Yes	1 (1.0%)	0 (0.0%)	1 (1.0%)	
**Procedure-related complications**				
No	61 (58.1%)	5 (55.6%)	56 (58.3%)	0.872
Yes	44 (41.9%)	4 (44.4%)	40 (41.7%)	

Continuous variables were presented as mean ± standard deviation with P-values derived from a two-sample t-test, or as median ± interquartile range (IQR) with P-values from a Kruskal-Wallis test. Categorical data were presented as frequency with percentages, and P-values were calculated using a Pearson Chi-square test.

Supplementary Table 11. Pre-treatment information about patients with BBA-ICA aneurysms

	Total	Surgery	EVT	P-value
**N**	19 (100.0%)	8 (42.1%)	11 (57.9%)	
**Age**	50.1 ± 10.3	47.1 ± 12.6	52.3 ± 8.2	0.296
**Sex**				
Female	10 (52.6%)	5 (62.5%)	5 (45.5%)	0.463
Male	9 (47.4%)	3 (37.5%)	6 (54.5%)	
**Arterial hypertension**				
No	11 (57.9%)	6 (75.0%)	5 (45.5%)	0.198
Yes	8 (42.1%)	2 (25.0%)	6 (54.5%)	
**Diabetes mellitus**				
No	17 (89.5%)	7 (87.5%)	10 (90.9%)	0.811
Yes	2 (10.5%)	1 (12.5%)	1 (9.1%)	
**Smoking**				
Never smoked	6 (40.0%)	1 (20.0%)	5 (50.0%)	0.072
Unknown	0 (0.0%)	0 (0.0%)	0 (0.0%)	
Previous smoker	3 (20.0%)	0 (0.0%)	3 (30.0%)	
Current smoker	6 (40.0%)	4 (80.0%)	2 (20.0%)	
**Hunt & Hess**				
1	1 (5.3%)	0 (0.0%)	1 (9.1%)	0.032
2	5 (26.3%)	3 (37.5%)	2 (18.2%)	
3	7 (36.8%)	1 (12.5%)	6 (54.5%)	
4	4 (21.1%)	4 (50.0%)	0 (0.0%)	
5	2 (10.5%)	0 (0.0%)	2 (18.2%)	
**Intracerebral hematoma (ICH)**				
None	15 (78.9%)	6 (75.0%)	9 (81.8%)	0.937
ICH <2 cm	2 (10.5%)	1 (12.5%)	1 (9.1%)	
ICH 2-5 cm	2 (10.5%)	1 (12.5%)	1 (9.1%)	
**Modified Fisher**				
1	2 (10.5%)	2 (25.0%)	0 (0.0%)	0.153
3	12 (63.2%)	5 (62.5%)	7 (63.6%)	
4	5 (26.3%)	1 (12.5%)	4 (36.4%)	
**Le Roux score**				
Le Roux score < 8	15 (78.9%)	7 (87.5%)	8 (72.7%)	0.435
Le Roux score ≥ 8	4 (21.1%)	1 (12.5%)	3 (27.3%)	
**Aneurysm size (mm)**	2.0 [2.0 - 3.0]	2.0 [2.0 - 2.0]	2.5 [2.0 - 3.0]	0.175
**Acute subdural hematoma**				
No	18 (94.7%)	8 (100.0%)	10 (90.9%)	0.381
Yes	1 (5.3%)	0 (0.0%)	1 (9.1%)	
**Rebleed**				
No	14 (77.8%)	5 (62.5%)	9 (90.0%)	0.163
Yes	4 (22.2%)	3 (37.5%)	1 (10.0%)	
**Aneurysm group**				
BBA-ICA	19 (100.0%)	8 (100.0%)	11 (100.0%)	.
**Hours from arrival to repair**	12.9 [7.5 - 18.1]	9.8 [1.6 - 15.7]	15.2 [10.8 - 32.1]	0.117
**Hours from ictus to repair**	23.9 [15.8 - 48.8]	20.7 [12.1 - 26.2]	23.9 [16.0 - 118.9]	0.386
**≥12 hours from ictus to repair**				
No	3 (15.8%)	2 (25.0%)	1 (9.1%)	0.348

Yes	16 (84.2%)	6 (75.0%)	10 (90.9%)	

Continuous variables were presented as mean ± standard deviation with P-values derived from a two-sample t-test, or as median ± interquartile range (IQR) with P-values from a Kruskal-Wallis test. Categorical data were presented as frequency with percentages, and P-values were calculated using a Pearson Chi-square test.

Supplementary Table 12. Outcome of patients with BBA-ICA blister aneurysms

	Total	Surgery	EVT	P-value
**N**	19 (100.0%)	8 (42.1%)	11 (57.9%)	
**Months after ictus**	7.5 ± 4.7	9.4 ± 5.6	6.1 ± 3.6	0.128
**Modified Rankin Score (mRS)**				
1	9 (47.4%)	2 (25.0%)	7 (63.6%)	0.277
2	6 (31.6%)	4 (50.0%)	2 (18.2%)	
3	1 (5.3%)	0 (0.0%)	1 (9.1%)	
5	1 (5.3%)	1 (12.5%)	0 (0.0%)	
6	2 (10.5%)	1 (12.5%)	1 (9.1%)	
**Radiological brain infarction**				
Absent	8 (42.1%)	2 (25.0%)	6 (54.5%)	0.198
Present	11 (57.9%)	6 (75.0%)	5 (45.5%)	
**Days of hospital stay**	24.5 ± 16.2	25.0 ± 17.3	24.2 ± 16.2	0.915
**CSF drainage (EVD and/or LD)**				
No	1 (5.3%)	1 (12.5%)	0 (0.0%)	0.228
Yes	18 (94.7%)	7 (87.5%)	11 (100.0%)	
**Tracheotomy**				
No	14 (73.7%)	5 (62.5%)	9 (81.8%)	0.345
Yes	5 (26.3%)	3 (37.5%)	2 (18.2%)	
**Hemicraniectomy**				
No	17 (89.5%)	6 (75.0%)	11 (100.0%)	0.080
Yes	2 (10.5%)	2 (25.0%)	0 (0.0%)	
**Procedure-related complications**				
No	15 (78.9%)	4 (50.0%)	11 (100.0%)	0.008
Yes	4 (21.1%)	4 (50.0%)	0 (0.0%)	

Continuous variables were presented as mean ± standard deviation with P-values derived from a two-sample t-test, or as median ± interquartile range (IQR) with P-values from a Kruskal-Wallis test. Categorical data were presented as frequency with percentages, and P-values were calculated using a Pearson Chi-square test.
